# Extracellular Vesicles in Osteoarthritis: From Pathogenic Mediators to Engineered Therapeutics in a Precision Medicine Roadmap

**DOI:** 10.1002/jex2.70131

**Published:** 2026-04-28

**Authors:** Duc‐Hiep Bach, Van Giang Bui, Thanh Liem Nguyen

**Affiliations:** ^1^ Vinmec Research Institute of Stem Cell and Gene Technology, College of Health Sciences VinUniversity Vinhomes Ocean Park Hanoi Vietnam; ^2^ Department of Radiology International Vinmec Hospital Hanoi Vietnam; ^3^ College of Health Sciences VinUniversity Vinhomes Ocean Park Hanoi Vietnam

## Abstract

Osteoarthritis (OA) remains without disease‐modifying sssstherapies, in part due to biological heterogeneity and a hostile joint microenvironment that undermines one‐size‐fits‐all interventions. Extracellular vesicles (EVs) play a dual role in OA pathophysiology: endogenous EVs disseminate pro‐inflammatory and catabolic signals that propagate cartilage degeneration, whereas therapeutic EVs most commonly derived from regenerative cell sources can deliver anti‐inflammatory and anabolic cues. We frame this contrast as the EV paradox and argue that it represents a central translational challenge explaining why robust preclinical efficacy has not yet translated into consistent clinical benefit.

We synthesize current evidence on EV biology in joint tissues, outcomes across preclinical models, and early human studies that demonstrate safety but limited efficacy. This analysis highlights key barriers to translation, including impaired EV function within inflamed and mechanically active joints, rapid clearance and limited tissue targeting, mismatch between animal models and human disease, and insufficient standardization of EV potency. Building on these insights, we propose a precision‐medicine roadmap that emphasizes patient stratification, rational EV design, improved delivery strategies, and manufacturing frameworks linked to mechanism‐anchored endpoints. Together, this framework reframes the EV paradox from a translational obstacle into a design principle for developing disease‐modifying EV‐based therapies for OA.

**Abbreviations**
ADAMTS: a disintegrin and metalloprotease with thrombospondin motifs
AUC: area under the curve
BML: bone marrow lesion
CMC: chemistry‐manufacturing‐controls
CQAs: critical quality attributes
DMM: destabilization of the medial meniscus
dGEMRIC: delayed gadolinium‐enhanced magnetic resonance imaging of cartilage
EV(s): extracellular vesicle(s)
GMP: good manufacturing practice
HA: hyaluronic acid
IA: intra‐articular
IL‐1β: interleukin‐1 beta
iPSC: induced pluripotent stem cell
KOOS: Knee Injury and Osteoarthritis Outcome Score
MIA: mono‐iodoacetate
MISEV: Minimal Information for Studies of Extracellular Vesicles
miRNA: microRNA
MMP: matrix metalloproteinase
MSC: mesenchymal stromal cell
NF‐κB: nuclear factor kappa B
OA: osteoarthritis
ARSI: Osteoarthritis Research Society International
PD: pharmacodynamic
PET: positron emission tomography
PK: pharmacokinetic
PNIPAM: poly(*N*‐isopropylacrylamide)
QC: quality control
RCT: randomized controlled trial
ROS: reactive oxygen species
SEC: size‐exclusion chromatography
SIRT1: sirtuin 1
SOX9: SRY‐box transcription factor 9
STV: synovial tissue volume
TFF: tangential‐flow filtration
TNF‐α: tumor necrosis factor alpha
TLR: Toll‐like receptor
VEGFA: vascular endothelial growth factor A
WOMAC: Western Ontario and McMaster Universities Osteoarthritis Index.

## 1 Introduction

Osteoarthritis (OA) is the most common joint disorder worldwide and a leading cause of disability in aging populations. Despite its enormous burden on quality of life and healthcare systems, no disease‐modifying therapies for OA have yet been approved. Current management ranging from lifestyle modification and analgesics to intra‐articular corticosteroids or hyaluronic acid remains purely symptomatic, delaying but not preventing progression to end‐stage joint replacement. This therapeutic void highlights a fundamental mismatch between existing interventions and the complex biology of OA (Hunter and Bierma‐Zeinstra 2019; Tang et al. 2025).

Traditionally regarded as a ‘wear‐and‐tear’ condition characterized by passive cartilage erosion, OA is now recognized as a heterogeneous, whole‐joint disease. Distinct molecular endotypes including inflammatory, bone‐remodelling, cartilage‐dominant, and metabolic phenotypes interact with systemic and biomechanical factors to drive joint degeneration. Although these endotypes are increasingly supported by transcriptomic, imaging, and biomarker studies, they should currently be viewed as mechanistic stratification frameworks rather than formally validated clinical subgroups. Individual patients often exhibit overlapping drivers, and standardized diagnostic criteria for endotype assignment have not yet been established. Consequently, endotyping is best interpreted as a biologically informed strategy for patient enrichment in trials rather than a definitive clinical classification (Hunter and Bierma‐Zeinstra 2019; Kraus et al. 2015; Mobasheri et al. 2017). Importantly, OA endotypes should not be interpreted as fixed or mutually exclusive patient categories. Individual joints commonly exhibit overlapping inflammatory, mechanical, metabolic, and bone‐remodelling processes, and the dominant pathogenic driver may shift during disease progression or treatment. Accordingly, endotypes are better viewed as dynamic mechanistic states rather than stable clinical labels. In this context, stratification is intended to enrich biological signal within trials and to guide hypothesis‐driven therapy selection, rather than to permanently classify patients. Pro‐inflammatory cytokines such as interleukin‐1β (IL‐1β) and tumor necrosis factor‐α (TNF‐α), together with catabolic enzymes including matrix metalloproteinases (MMPs) and a disintegrin and metalloprotease with thrombospondin motifs (ADAMTS), establish a hostile joint microenvironment that suppresses intrinsic repair and accelerates tissue degeneration (Tang et al. 2025; Martel‐Pelletier et al. 2016; Yao et al. 2023). This evolving understanding underscores the need for therapeutic strategies that are both targeted and adaptable.

Within this landscape, extracellular vesicles (EVs) have emerged as a particularly compelling yet paradoxical class of biological mediators. The label ‘exosomes’ was common in early regenerative literature, but current understanding and the 2023 ISEV guidelines (Minimal Information for Studies of Extracellular Vesicles [MISEV] 2023) emphasize that preparations from cell cultures or fluids represent a heterogeneous mix of small EVs, including exosomes (30–150 nm) and microvesicles (100–1000 nm) with overlapping cargos and markers (Welsh et al. 2024). Because no current isolation method (e.g., ultracentrifugation, tangential‐flow filtration, size‐exclusion chromatography [SEC]) can perfectly separate these subtypes, and both contribute to biological signalling, the umbrella term EVs is now preferred (van Niel et al. 2018). This terminology avoids over‐specific claims, aligns with translational and regulatory expectations, and reflects the likelihood that functional efficacy in OA arises from integrated vesicular cargo rather than a single vesicle class (Xu et al. 2025).

Endogenous EVs released by diseased joint tissues (e.g.,, chondrocytes, synoviocytes, and osteocytes/subchondral bone) can propagate inflammatory and degradative signals, amplifying OA pathology, whereas therapeutic EVs especially those derived from mesenchymal stromal cells (MSCs) or induced pluripotent stem cells (iPSCs)‐derived MSCs can deliver anti‐inflammatory mediators, anabolic growth factors, and microRNAs that promote cartilage homeostasis and repair (Bertolino et al. 2023; Ni et al. 2020; Wu et al. 2022). Although MSCs have demonstrated safety and symptomatic benefit in small‐scale OA studies, large trials often fail to show sustained structural regeneration. This underperformance reflects several recurring mechanistic limitations. (1) Product heterogeneity: differences in donor source, cell passage, culture conditions and epigenetic drift lead to variable secretome potency and lot‐to‐lot inconsistency (Copp et al. 2023). (2) Hostile joint microenvironment: inflammatory cytokines, oxidative stress, low pH and proteolytic enzymes quickly diminish cell viability and reprogram their paracrine output (Sun et al. 2025). (3) Poor persistence and targeting: injected MSCs are cleared rapidly by synovial washout or immune surveillance, with negligible retention in cartilage or subchondral bone (Copp et al. 2023). (4) Manufacturing & translational constraints: large‐scale expansion can induce senescence, reduced potency, genetic instability, and regulatory hurdles for reproducible dosing (José Alcaraz 2024, Klyucherev et al. 2025). Together, these barriers help explain the inconsistency of whole‐cell MSC therapy across OA subtypes, motivating a shift toward more standardizable, subcellular effectors EVs as a next‐generation therapeutic platform (Sun et al. 2025). Importantly, EVs are not simply a smaller version of MSC therapy but a mechanistically distinct modality. Many limitations of MSC transplantation arise from the need for living cells to survive and function within an inflamed joint environment. EVs, by contrast, act as cell‐free signalling entities whose activity depends on molecular cargo rather than cellular persistence (Welsh et al. 2024). Consequently, they do not require engraftment, can be administered repeatedly, and can be standardized and quality‐controlled as biological products. Thus, EVs isolate the paracrine effector function of MSCs while avoiding several viability and manufacturing constraints inherent to whole‐cell transplantation. However, these advantages do not eliminate translational challenges and in some cases introduce new ones. Increasing therapeutic sophistication such as defined cargo loading, surface targeting modifications, conditioning strategies, biomaterial depots, or combination regimens can improve biological specificity but simultaneously increases manufacturing complexity, quality‐control requirements, and regulatory scrutiny. Each additional design layer creates new sources of variability in potency, stability, and batch reproducibility, and may complicate scalability and cost‐effective deployment. Accordingly, the goal of EV engineering should not be maximal biological complexity, but controllable and mechanism‐anchored simplicity: interventions should incorporate only those modifications necessary to achieve a measurable pharmacodynamic effect. This balance between biological precision and manufacturability represents a central constraint in translating EVs from experimental therapeutics to practical clinical medicines. This duality EVs as both pathogenic messengers and therapeutic agents forms what we term the EV paradox, framing a central challenge and opportunity in the field.

The present review synthesizes evidence on the dual role of EVs in OA, critically examines why striking preclinical successes have not yet yielded consistent clinical efficacy, and proposes a precision‐medicine roadmap to overcome these barriers. We argue that resolving the EV paradox will require aligning EV design, delivery, and patient selection with disease endotypes and translational constraints, rather than relying on one‐size‐fits‐all approaches, to transform EVs into truly disease‐modifying therapeutics (Bertolino et al. 2023).

## 2 Part I. The Dual Nature of Extracellular Vesicles in OA

### 2.1 Endogenous EVs as Pathogenic Messengers

EVs are small lipid bilayer particles including exosomes and microvesicles that carry regulatory cargo (microRNAs, proteins, and lipids) and act as organized mediators of intercellular communication rather than inert debris (Welsh et al. 2024; Bertolino et al. 2023). Within the osteoarthritic joint, EVs are released by virtually all tissue compartments, including chondrocytes, synovial fibroblasts, osteoblasts and osteoclasts, endothelial cells, and infiltrating immune cells (Bertolino et al. 2023; Boere et al. 2018). In a healthy state, such vesicular signalling supports tissue homeostasis; however, under the stresses of OA mechanical overload, chronic low‐grade inflammation, oxidative injury, and aging EV cargo becomes skewed toward disease‐promoting instructions (Bertolino et al. 2023; Mao et al. 2018). Importantly, EV‐mediated communication operates alongside, rather than replacing, classical signalling modalities in OA. Joint tissues exchange information through soluble cytokines and chemokines, growth factors, and matrix‐derived damage‐associated molecular patterns released during cartilage breakdown (Scanzello and Goldring 2012). However, these signals primarily act through concentration‐dependent diffusion and are rapidly degraded in the synovial environment (Scanzello and Goldring 2012; Yáñez‐Mó et al. 2015). EVs differ in that they encapsulate coordinated sets of RNAs, proteins, and lipids within a protective membrane, enabling protected transport, simultaneous delivery of multiple signals, and cell‐type‐specific uptake (Yáñez‐Mó et al. 2015; Tkach and Théry 2016). Thus, EVs should not be viewed as the dominant signalling pathway in OA, but as an organized communication layer capable of integrating and propagating inflammatory or reparative programs across joint compartments.

Multiple pathogenic processes can be attributed to this shift. Inflammatory amplification arises when EVs derived from inflamed synovium and stressed chondrocytes deliver cytokines, chemokines, and miRNAs that activate NF‐Κb (nuclear factor kappa B) pathways in recipient cells. This maintains synovitis, drives macrophages toward a pro‐inflammatory phenotype, and upregulates catabolic enzymes (Ni et al. 2020; Wu et al. 2022; Cosenza et al. 2018). Other vesicles foster matrix degradation and chondrocyte dysfunction by stimulating metalloproteinases and aggrecanases, reducing anabolic synthesis, and inducing apoptosis or senescence (Bertolino et al. 2023; Liu et al. 2023). Crosstalk between cartilage and subchondral bone is mediated by EVs; in particular, osteoclast‐derived exosomes (e.g., those enriched in miR‐214‐3p) impair osteoblast function and are linked to abnormal subchondral bone remodelling (Li et al. 2016), while subchondral‐bone‐derived exosomes (e.g., those carrying miR‐210‐5p) drive catabolic reprogramming in chondrocytes (Bertolino et al. 2023; Wu et al. 2021). With aging, senescent cells accumulate in joint tissues; EV output from senescent chondrocytes increases and correlates with the senescent‐cell burden, and these vesicles transfer senescence markers to bystander cells, propagating senescence and impairing cartilage regeneration mechanisms linked to age‐related OA progression and to inflammatory programs driven by senescence/SASP cross‐talk the joint (Han et al. 2024). Collectively, these findings position endogenous EVs as active conveyors of disease, orchestrating joint‐wide degeneration rather than passive by‐products of tissue breakdown.

### 2.2 Therapeutic and Engineered EVs as Regenerative Mediators

In sharp contrast, EVs harvested from regenerative sources demonstrate immunomodulatory and reparative properties. Mesenchymal stromal cell–derived EVs (MSC‐EVs) are the most widely studied and have been shown to suppress synovial inflammation, promote reparative (M2) macrophage polarization, and reduce pro‐catabolic cytokines in OA models (Bertolino et al. 2023; Ni et al. 2020; Zhang et al. 2020). These reparative effects are mediated by vesicular molecular cargo, as detailed in the mechanistic axes section below (Tao et al. 2017; Wu et al. 2019). Beyond cartilage, therapeutic EVs influence angiogenic/vascular signalling (e.g., via VEGFA targeting) (Liu et al. 2022) and attenuate aberrant subchondral bone remodelling, underscoring system‐level relevance to joint integrity. Importantly, EVs possess drug‐like malleability: parent cells can be preconditioned and genetically modified to enrich vesicle cargo (miRNAs, proteins) and/or display targeting ligands, yielding ‘designer EVs’ tailored to counteract defined drivers such as excessive inflammation or osteoclast‐biased bone turnover.

Despite their therapeutic promise, engineered EVs face substantial challenges related to large‐scale manufacturing, cost, and reproducibility. Clinical translation will require scalable, GMP (Good manufacturing practice)‐compatible production platforms, including closed‐system bioreactor expansion and high‐throughput purification methods such as tangential‐flow filtration, to enable consistent yield and quality at reduced cost. Equally important is the adoption of mechanism‐anchored potency assays and standardized release criteria to limit batch‐to‐batch variability, particularly for engineered or cargo‐modified EVs (Welsh et al. 2024; Wu et al. 2024; Estes et al. 2022). Together, these manufacturing disciplines are likely to determine whether engineered EVs can progress from bespoke laboratory products to economically viable therapeutic modalities.

### 2.3 The EV Paradox as a Unifying Concept

These contrasting activities establish what we term the EV paradox: vesicles in OA are simultaneously pathogenic messengers and therapeutic agents. The joint can be conceptualized as a biological arena in which disease‐propagating EVs compete against restorative signals delivered through therapy. This paradox offers a coherent explanation for otherwise puzzling observations. For instance, osteoclast‐ and osteocyte‐derived EVs that drive catabolic programs in chondrocytes while therapeutic EVs (notably MSC‐EVs) exert reparative and immunomodulatory effects in preclinical joint models (Bertolino et al. 2023; Liu et al. 2021; Liu et al. 2025).

Because the same vesicle architecture can mediate either disease propagation or tissue repair, a structured comparison is required. We therefore summarize the defining biological and functional differences between pathogenic endogenous EVs and therapeutic MSC‐derived EVs in **Table** **1**. Endogenous EVs released from diseased joint tissues are enriched in pro‐inflammatory, catabolic, and senescence‐associated cargo that amplifies cartilage breakdown, synovitis, and abnormal subchondral bone remodelling. In contrast, therapeutic MSC‐derived EVs consistently carry anti‐inflammatory and chondroprotective microRNAs and proteins that promote macrophage reprogramming, cartilage anabolism, and tissue homeostasis. This side‐by‐side comparison highlights that the EV paradox is fundamentally a cargo‐logic problem: the same vesicular architecture can encode either disease‐propagating or disease‐antagonistic instructions depending on its molecular payload.

**Table 1:** Comparative biological properties and functional consequences of pathogenic endogenous EVs versus therapeutic MSC‐derived EVs in osteoarthritis (Welsh et al. 2024; Mao et al. 2018; Tao et al. 2017, Wu et al. 2019; Liu et al. 2025; Jeon et al. 2019; Chen et al. 2018).

Feature	Pathogenic endogenous EVs (OA joint‐derived)	Therapeutic MSC‐derived EVs
**Primary source**	Senescent joint cell; subchondral bone osteoblast lineage EVs	Synovial MSCs; infrapatellar fat pad MSCs; engineered MSC‐EVs
**Representative miRNA cargo**	Pro‐degenerative EV miRNAs reported in OA bone–cartilage crosstalk (representative example: miR‐23b‐3p)	miR‐140‐5p; miR‐100‐5p; miR‐92a‐3p (engineered or enriched)
**Dominant signalling effect**	Catabolic and senescence‐associated signalling	Anti‐catabolic and pro‐chondrogenic signalling
**Primary target cells**	Chondrocytes (osteochondral unit)	Chondrocytes; joint‐resident cells
**Net biological outcome**	Promotion of OA progression	Attenuation of cartilage degeneration in OA models
**Therapeutic implication**	Disease‐propagating signals; not directly targetable	Programmable biologic delivery vehicles
**Key translational limitations**	Reflect disease state rather than a therapeutic product	Heterogeneity, potency definition, standardization

This mechanistic contrast establishes the basis for the EV paradox and provides the foundation for translating EV biology into therapeutic design.

This duality helps reconcile a common pattern: robust preclinical benefits versus early human evidence that is so far limited and has not established consistent clinical efficacy. Patient‐level and stage‐dependent heterogeneity in OA biology and EV cargo further argues against one‐size‐fits‐all products. Finally, without targeting/retention to deep cartilage and subchondral bone, injected vesicles face rapid clearance and poor intra‐cartilage exposure; by contrast, platforms that penetrate cartilage or prolong joint residence improve intra‐tissue delivery and therapeutic readouts in models (Geiger et al. 2018; Joshi et al. 2025; Bajpayee and Grodzinsky 2017).

**Figure 1:**
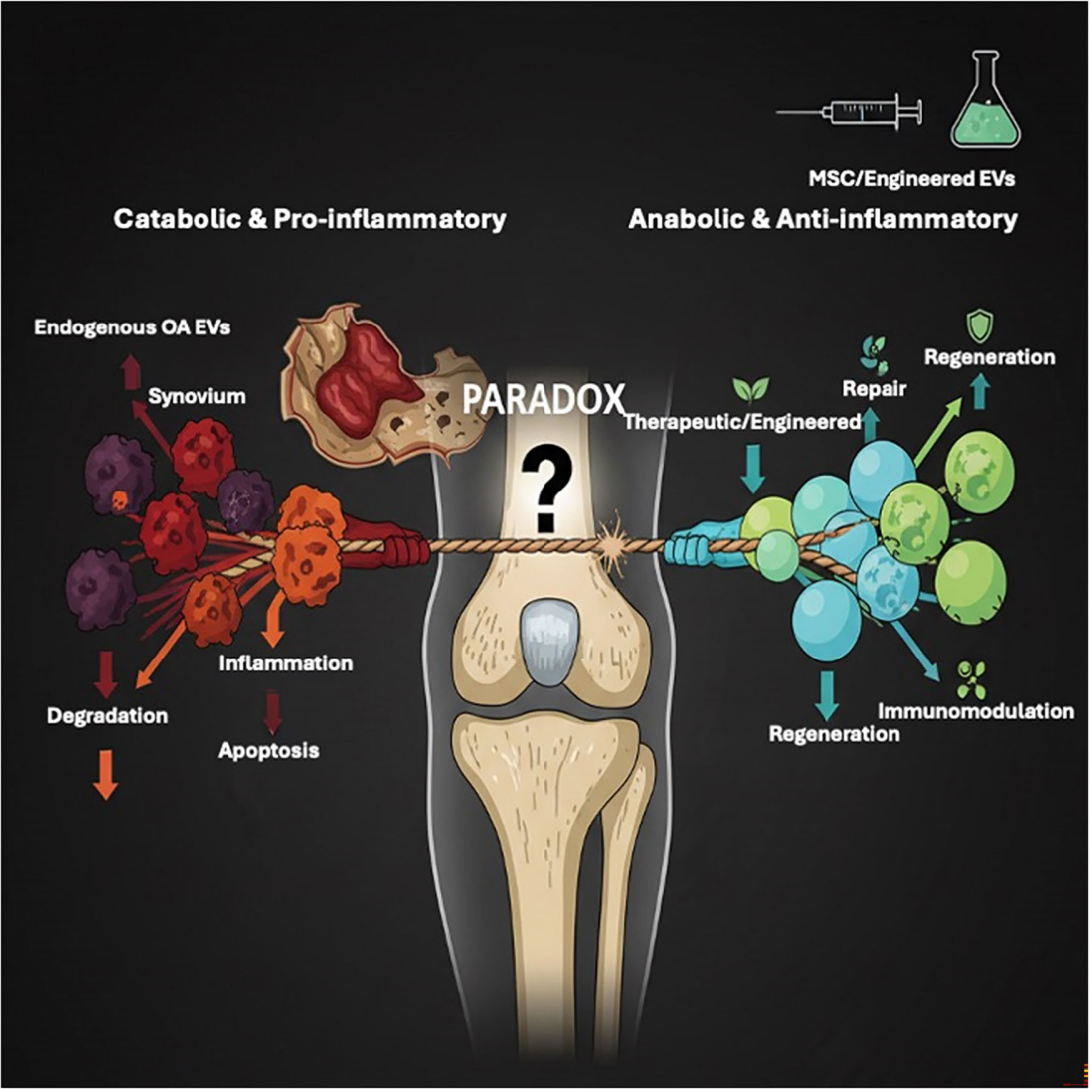
The EV paradox in osteoarthritis: pathogenic versus therapeutic signallingExtracellular vesicles (EVs) embody a central paradox in osteoarthritis (OA). On the one hand, endogenous OA‐derived EVs released from synovium, cartilage, and bone compartments propagate catabolic and pro‐inflammatory programs driving matrix degradation, inflammation, and apoptosis. On the other hand, therapeutic or engineered EVs (typically derived from mesenchymal stromal cells) can deliver anti‐inflammatory and anabolic instructions, supporting immunomodulation, tissue repair, and regeneration. The balance between these opposing vesicular signals shapes whether the joint progresses toward degeneration or recovery. This paradox helps explain why robust preclinical efficacy has not yet translated into consistent clinical benefit and highlights the need for precision design of EV therapies with optimized cargo, surface targeting, and delivery kinetics.

This duality is captured in the EV paradox, where the same class of vesicles can either propagate joint degeneration or deliver reparative cues, depending on their origin and design (**Figure** **1**).

**Figure 2:**
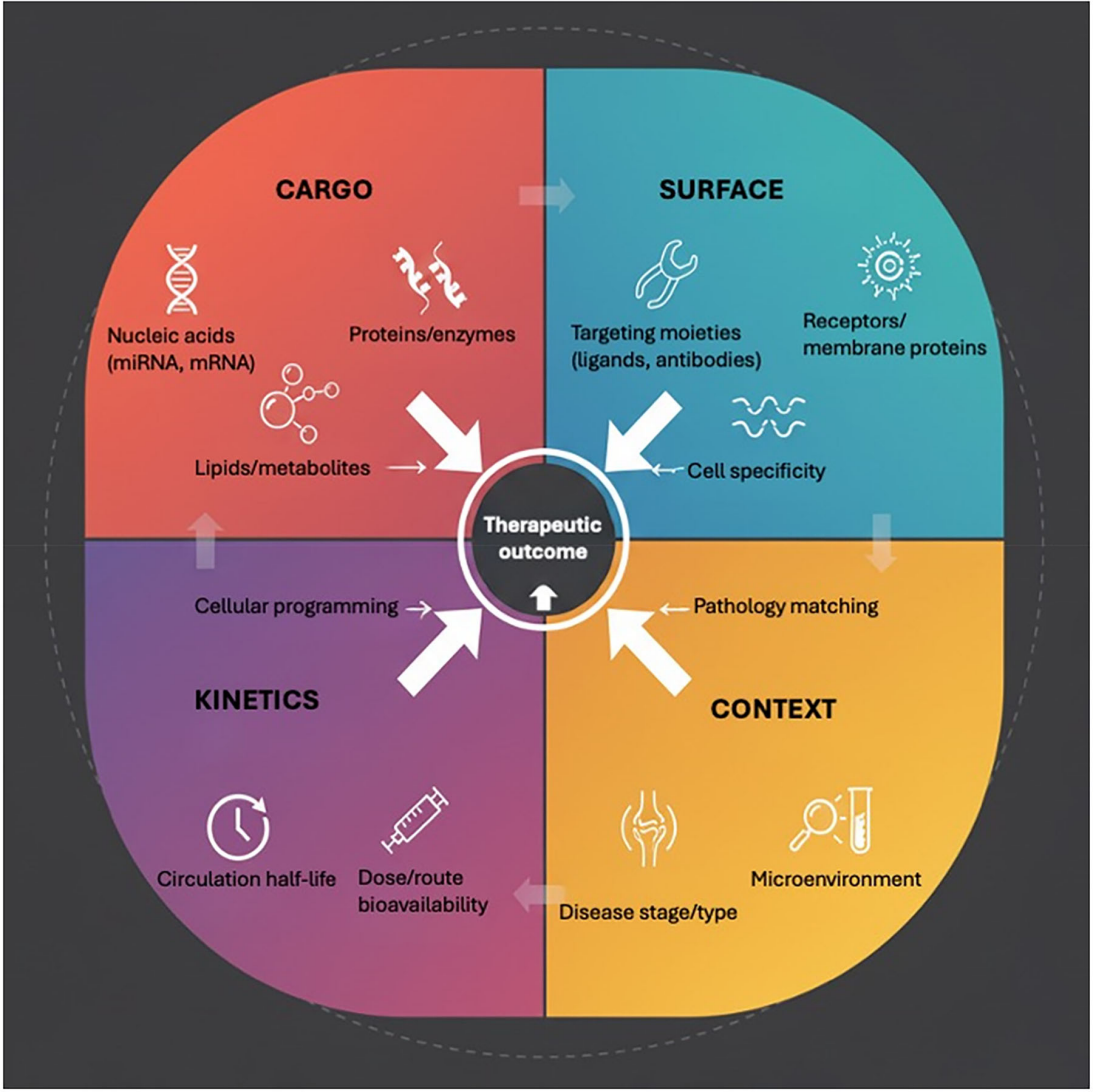
Mechanistic axes shaping extracellular vesicle outcomes in osteoarthritis. Therapeutic outcomes of extracellular vesicles (EVs) are governed by four interdependent mechanistic axes. Cargo encompasses nucleic acids, proteins, and lipids/metabolites that encode specific biological signals. Surface properties, including membrane receptors and engineered targeting moieties, dictate tissue tropism and cell specificity. Kinetics reflects circulation half‐life, route of delivery, bioavailability, and strategies for sustained exposure. Context includes the disease stage, type, and microenvironment, which collectively influence whether EVs are received as regenerative or pathogenic messages. Together, these axes provide a framework for designing next‐generation EV therapeutics capable of achieving disease‐modifying effects in osteoarthritis.

To resolve the EV paradox, it is helpful to consider the four mechanistic axes cargo, surface, kinetics, and context that together determine whether vesicles drive degeneration or repair (**Figure** **2**).

### 2.4 Mechanistic Axes Shaping EV Outcomes

To resolve this paradox, it is helpful to consider four mechanistic axes that dictate whether EVs drive degeneration or repair. The first is cargo composition: pathogenic EVs are enriched in catabolic signals, whereas therapeutic MSC‐EVs must deliver antagonistic miRNAs/proteins that re‐engage chondrogenic programs and damp pro‐inflammatory pathways (e.g., NF‐ κB); miRNA‐enriched MSC‐EVs (miR‐100‐5p), miR‐140‐5p) restore cartilage structure and homeostasis in vivo (Bertolino et al. 2023; Wu et al. 2019; Tao et al. 2017). Building on this concept, specific EV cargo components have been functionally validated in knee OA models. Among EV cargo molecules, two microRNAs emerge as repeatedly validated functional mediators: miR‐140‐5p and miR‐100‐5p. EVs enriched in miR‐140‐5p consistently prevent cartilage degeneration and support chondrocyte anabolism in vivo, whereas miR‐100‐5p–containing EVs demonstrate cartilage‐protective activity and functional improvement in experimental OA models (Tao et al. 2017; Wu et al. 2019). Together these define a core chondroprotective EV cargo signature. Additional candidates including miR‐146a, miR‐23/27, miR‐95‐5p and miR‐92a‐3p show supportive evidence but remain context‐dependent modulators rather than primary effectors (Mao et al. 2018; Guan et al. 2018; Wang et al. 2024; Chai et al. 2022; Mao et al. 2018; Akhtar et al. 2010). These observations provide a rationale for engineering EVs with defined cargo profiles for OA therapy. The second is surface biology: vesicle tropism is shaped by membrane proteins (tetraspanins/integrins) and can be enhanced by engineering (e.g., charge‐reversed or peptide‐modified EVs) to target chondrocytes and joint tissues (van Niel et al. 2018; Hoshino et al. 2015; Zhang et al. 2024). The third axis is dose and time: OA is a chronic disease, and single bolus intra‐articular delivery is typically insufficient because solutes/particles clear rapidly from the joint and penetrate cartilage poorly hence the need for sustained exposure via repeated dosing or retention platforms that increase intra‐cartilage availability (Geiger et al. 2018; Joshi et al. 2025; Bajpayee and Grodzinsky 2017). Finally, the joint environment itself strongly influences therapeutic efficacy. For instance, the joint milieu such as the inflamed joint microenvironment (cytokines/ROS, local acidity and proteases, especially across cartilage‐bone units) modulates EV integrity and uptake (including formation of a protein “corona”) and can blunt therapeutic signalling motivating strategies that pre‐condition the milieu or co‐deliver EVs with environment‐modulating carriers (Yao et al. 2023; Tóth et al. 2021; Hu et al. 2021).

### 2.5 Conceptual Model for Therapeutic Development

Viewing OA as an EV‐mediated network disorder reframes therapeutic goals: endogenous vesicles encode and propagate catabolic programs in cartilage–bone–synovium units, whereas engineered therapeutic vesicles can be designed as programmable countersignals. Translation thus hinges on three interdependent variables: (i) cargo potency: vesicles must deliver disease‐antagonistic payloads (e.g., miRNA‐enriched EVs that restore chondrogenic balance and suppress inflammatory signalling; (ii) precision of tissue targeting: native tetraspanins/integrins and engineered ligands govern tropism to chondrocytes, synoviocytes, or subchondral bone; and (iii) persistence of signal in a receptive environment overcoming rapid joint clearance and poor cartilage penetration via retention/targeting platforms. Framed through this EV paradox, past preclinical‐clinical gaps become explicable and the actionable levers cargo, surface, kinetics, and context emerge as the knobs to optimize for bona fide disease‐modifying efficacy (Bertolino et al. 2023; Liu et al. 2021; Liu et al. 2025; Geiger et al. 2018).

## 3 Part II. The Translational Paradox: Why Experimental Potency Becomes Clinical Modesty

**Preclinical signal (animal models)**. Across rodent and large‐animal models of OA, EVs derived from regenerative sources consistently blunt synovitis, protect cartilage structure, and improve readouts of joint function (Bertolino et al. 2023; Ni et al. 2020). Intra‐articular EV administration mitigates proteoglycan loss and collagen disorganization, suppresses catabolic transcriptional programs in chondrocytes, and reduces macrophage‐driven inflammation in synovium (Bertolino et al. 2023; Ni et al. 2020; Tao et al. 2017; Wu et al. 2019). Histology typically registers lower Osteoarthritis Research Society International (OARSI) grades in treated joints, while micro‐computed tomography or *ex vivo* biomechanics occasionally show partial normalization of subchondral architecture and cartilage stiffness (Bertolino et al. 2023; Ni et al. 2020). Gait analysis and weight‐bearing assays frequently confirm a functional signal that aligns with the structural changes (Ni et al. 2020).

These observations are mechanistically coherent with EV cargo. Vesicles harvested from MSCs or closely related progenitors carry miRNAs that restrain NF‐κB signalling and aggrecanases, proteins that promote autophagy, oxidative‐stress control, and survival pathways, lipids that modulate membrane‐proximal signalling in recipient cells (van Niel et al. 2018; Tao et al. 2017; Wu et al. 2019). In co‐culture systems, these vesicles re‐program synovial macrophages toward reparative states, attenuate fibroblast‐like synoviocyte activation, and restore anabolic balance in chondrocytes (Bertolino et al. 2023; Ni et al. 2020). Head‐to‐head and comparative studies in some models suggest that when dose and exposure are optimized, vesicles recapitulate a substantial fraction of the chondroprotection historically attributed to their parent cells supporting the view that paracrine (including vesicular) signalling is a major effector arm of cell therapy in OA (Bertolino et al. 2023; Ni et al. 2020).

The durability and magnitude of the preclinical signal nevertheless track with disease context. Acute or subacute models anterior cruciate ligament transection (ACLT), destabilization of the medial meniscus (DMM), or mono‐iodoacetate (MIA) generate high‐incidence lesions in young animals whose joint microenvironments are inflamed but comparatively plastic; in these settings, single‐bolus dosing is often sufficient to shift short‐term histology, imaging surrogates, and pain behaviours (Ni et al. 2020). When experiments are extended to older animals, longer time frames, or conditions that incorporate metabolic stress and low‐grade systemic inflammation, effect sizes commonly attenuate as an early warning that EVs are most potent where the disease network is least entrenched and the joint remains biologically receptive (Ni et al. 2020).

Several variables inside the ‘preclinical success’ umbrella deserve emphasis because they foreshadow translational failure models. First, dose is commonly defined by particle counts or protein mass, metrics that are agnostic to mechanism; two preparations with identical counts can differ markedly in biological potency (Welsh et al. 2024). Second, vesicle composition depends on the state of the parent cell and the culture conditions used to produce them; subtle shifts in oxygen tension, cytokine milieu, or passage history re‐write vesicle cargo (Welsh et al. 2024). Third, routes and schedules vary widely across studies, confounding comparisons: most investigations use a single intra‐articular bolus, but some deliver repeated doses or combine EVs with biomaterial scaffolds that extend residence time; delivery constraints (rapid clearance, poor cartilage penetration) motivate retention/targeting platforms to increase intra‐cartilage exposure (Geiger et al. 2018; Bajpayee and Grodzinsky 2017). When the preclinical record is read critically, it reveals robust biology and substantial heterogeneity in the way that biology is instantiated (Welsh et al. 2024; Bertolino et al. 2023).

**Systematic evidence from preclinical models**. Beyond individual reports, high‐impact reviews and systematic syntheses consistently demonstrate a robust therapeutic effect of EVs across animal models of OA. These analyses confirm significant improvements in cartilage integrity (OARSI histological scores), reduced synovial inflammation, and functional recovery, with standardized effect sizes in the moderate‐to‐large range (Ni et al. 2020; Liu et al. 2023). Seminal preclinical studies further show that EVs enriched with reparative miRNAs (e.g., miR‐140‐5p) reproducibly protect cartilage and prevent OA progression in vivo (Tao et al. 2017). Together, these findings provide a mechanistic anchor for translation, but also highlight the urgent need for standardized potency metrics to reduce variability between studies. Although the aggregate preclinical literature suggests a consistent therapeutic signal, it should be interpreted cautiously. Preclinical regenerative studies, including MSC and EV research, are susceptible to publication bias, small sample sizes, and methodological heterogeneity (Sena et al. 2010; Kilkenny et al. 2010). Neutral or negative animal experiments are less frequently reported, and many studies lack blinding, randomization, or predefined outcome criteria (Sena et al. 2010). Meta‐research analyses demonstrate that such biases systematically inflate apparent treatment effects and reduce reproducibility across laboratories (Sena et al. 2010; Kilkenny et al. 2010; Begley and Ellis 2012). Accordingly, strong preclinical efficacy should be interpreted as evidence of biological potential rather than proof of clinical effectiveness. Recognition of this bias helps explain the translational paradox in OA: robust activity in controlled experimental systems may not translate into consistent benefit in heterogeneous human disease.

**Clinical signal (safety, limited efficacy)**. The early clinical curve is characterized by feasibility and safety rather than efficacy. Intra‐articular administration of EV‐rich preparations has been well tolerated in small cohorts, with adverse events largely limited to transient local reactions; however, randomized, placebo‐controlled trials using single‐dose injection have not demonstrated superiority on standard outcomes pain, function, or composition MRI over mid‐term follow‐up (Bertolino et al. 2023; Ni et al. 2020). Uncontrolled pilot studies occasionally report symptomatic improvement, but the magnitude and persistence of these effects are difficult to disentangle from regression to the mean, placebo dynamics, background therapy use, and the natural variability of OA symptoms (Bertolino et al. 2023).

The gap between experimental potency and clinical modesty becomes clearer when one considers the constrains of first‐in‐human designs. Typical protocols enrol radiographically defined, heterogeneous grade II–III OA, restrict to a single injection to de‐risk safety, and select endpoints aligned with regulatory familiarity rather than mechanistic sensitivity. EV preparations are often specified by process (source, isolation) without potency assays to anchor dose selection. Concomitant therapies analgesics, physical therapy, intra‐articular hyaluronan or prior steroid exposure introduce noise that further dilutes the capacity of detect an EV‐specific signal. Summed across these factors, early trials demonstrate that EVs can be delivered safely to human joints; they elicit very little about whether the right vesicles reached the right cells for long enough to matter (Bertolino et al. 2023). Read this discrepancy not as a refutation of the biology, but as a calibration problem of what the vesicle carry, where they go, how long they stay, and the milieu they enter (Bertolino et al. 2023; Geiger et al. 2018; Bajpayee and Grodzinsky 2017).

**Figure 3:**
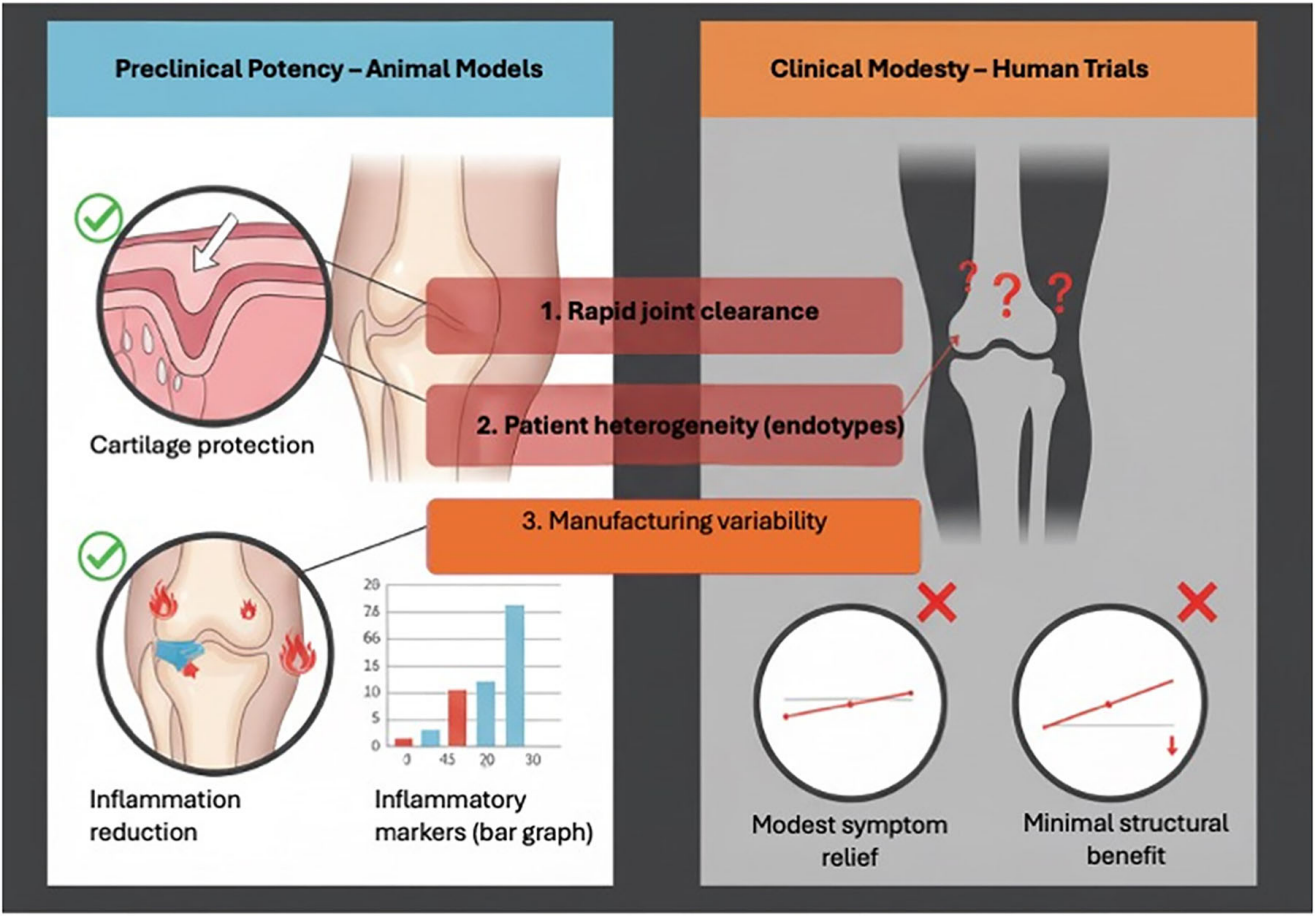
Translational gap: from preclinical potency to clinical modesty. Despite robust efficacy in animal models, including cartilage protection and inflammation reduction, clinical trials have so far demonstrated only modest symptom relief and minimal structural improvement. Key barriers to translation include rapid clearance of vesicles from large human joints, heterogeneity across patient endotypes, and variability in EV manufacturing.

Taken together, these observations highlight the translational gap where dramatic potency in animal models gives way to only modest outcomes in human trials driven by rapid clearance, patient heterogeneity, and manufacturing variability (**Figure** **3**).

Importantly, this translational gap also reflects fundamental pharmacokinetic and scaling mismatches between preclinical models and human joints. Rodent knees are orders of magnitude smaller and experience different mechanical loading and synovial turnover, allowing intra‐articularly delivered EVs to achieve higher effective concentrations and longer tissue exposure. In contrast, the large volume, dynamic mechanical forces, and efficient lymphatic clearance of human knee joints markedly reduce EV residence time and target exposure following bolus injection (Evans et al. 2014; Brown et al. 2006; Huang et al. 2022; Kou et al. 2022). Consequently, dosing regimens that appear effective in rodent models may substantially underdeliver therapeutic signals in humans, representing a key pharmacokinetic barrier to in vivo translation.

### 3.1 Mechanistic Axes Explaining The Gap (Cargo, Surface, Kinetics, Context)

Four interdependent axes cargo, surface, kinetics, and context convert that calibration problem into an actionable map.

**Cargo potency**. Vesicles are programmable messages, but clinical products are too often defined by how they are made rather than by what they do (Welsh et al. 2024; Bertolino et al. 2023). Without human cell‐based potency metrics suppression of inflammatory transcriptional programs in synoviocytes, restoration of anabolic signalling and autophagy in chondrocytes, normalization of osteoclastogenesis and osteoblast function ‘dose’ collapses to a particle count (Welsh et al. 2024). This is not a benign simplification: particle count does not capture the abundance or integrity of key miRNAs, the presence of inhibitory cargo, or the stoichiometry of proteins that execute the intended mecssssshanism (Welsh et al. 2024; Bertolino et al. 2023). Moreover, parent‐cell state imprints the message; hypoxic preconditioning or cytokine exposure can substantially change vesicle content (van Niel et al. 2018; Bertolino et al. 2023). Inconsistent cargo logic translates directly into inconsistent clinical biology. Beyond the message they carry (cargo), the destination the cells and micro‐anatomical niches EVs reach governs whether any message can be heard, which elevates the importance of surface biology.

**Surface targeting**. Where vesicles go is as important as what they carry. Native EVs exhibit limited penetration and uncertain tropism to deep‐zone chondrocytes and osteochondral units the micro‐anatomical niches that determine structural progression and pain (Bajpayee and Grodzinsky 2017; Hoshino et al. 2015). In rodents, millimetre‐scale diffusion distances and thin cartilage layers partly mask this limitation. In human knees, centimetre‐scale diffusion and dense extracellular matrix reduce the probability of stochastic arrival at the correct cell in sufficient numbers (Bajpayee and Grodzinsky 2017). Engineering surface ligands cartilage‐homing peptides, collagen‐binding motifs, or bone‐targeting moieties should increase effective dose at the site of pathology, but these features are rarely incorporated into first‐in‐human products (Bajpayee and Grodzinsky 2017; Hoshino et al. 2015). Targeting without time is insufficient; even perfectly aimed vesicles must persist long enough to matter, which makes kinetics and exposure the next limiting axis.

**Kinetics and exposure**. Synovial clearance and lymphatic drainage reduce intra‐articular vesicle concentrations rapidly, particularly in large joints (Bajpayee and Grodzinsky 2017). A single bolus, even if nominally ‘high dose’, is unlikely to deliver a biologically meaningful area‐under‐the‐curve (AUC) at the chondrocyte or subchondral bone interface. Accordingly, dosing must be planned for sustained intra‐articular exposure either by using a residence‐time depot or by pre‐specifying a repeat‐injection schedule outlined below. Because synovial turnover and lymphatic drainage clear solutes and nanoparticles from the joint within hours–days, clinically meaningful exposure usually requires either a retention depot or repeat dosing. Reviews and experiments in OA models show that hydrogels (including HA‐based) can prolong intra‐articular residence and exposure of EVs versus free bolus injections (van de Looij et al. 2023; Yang et al. 2021).

**Human signal to anchor expectations**. A randomized, triple‐blind trial in knee OA tested **a** single intra‐articular dose of placental MSC‐EVs (5 mL at 7 × 10^9^ particles/mL; ∼3.5 × 10^10^ total particles) safe, but with no clinical or MRI benefit over placebo at that one‐off exposure. This supports designing repeat dosing or depot strategies rather than relying on a single bolus (Bolandnazar et al. 2024).

### 3.2 Pragmatic Schedules To Pre‐Specify (Proposal)

- **Depot‐integrated EVs** (e.g., HA or thermoresponsive hydrogel): 1 IA injection every ∼4–8 weeks for 2–3 doses (induction), then maintenance every 3–6 months guided by PD (pharmacodynamic) markers and imaging; the residence‐time advantage of hydrogels underpins this cadence (van de Looij et al. 2023).
- **Free EVs** (no depot): plan repeat IA injections at 4–12‐week intervals (e.g., 0/4/12 weeks) with maintenance every 3–6 months if on‐target biology persists; preclinical syntheses indicate that repeated dosing outperforms single bolus, with weekly cadences common in small‐animal work (translation to humans still to be optimized) (van de Looij et al. 2023; Kong et al. 2024).

**How to report ‘dose’**. For cross‐study comparability, specify both particles and EV‐protein and pair dose with the mechanism‐anchored potency assay (e.g., NF‐κB reporter suppression, chondrocyte anabolism rescue), per MISEV2023 quantitative/functional guidance. Representative preclinical units include particle counts (e.g., ∼10^10^ particles per injection in rodent knees) and EV‐protein amounts (e.g., 25–50 µg per injection or ∼100 µg on weekly schedules in some models); use these only as illustrative reporting units and justify your final dose with potency and PK (pharmacokinetic).

Residence‐time technologies shear‐thinning or thermoresponsive hydrogels, viscous carriers, nanoparticulate depots are rational, preclinically supported answer, yet most clinical protocols have not integrated them as part of the therapeutic product (Geiger et al. 2018; Joshi et al. 2025). Where depots are not feasible, rational repeat dosing calibrated to joint volume, expected clearance, and early pharmacodynamicPD response is essential. The kinetics problem is not cosmetic; it is central. Yet exposure still competes with biology: a hostile joint can erase signals even when they arrive on time, placing the context of delivery at centre stage.

**Context and receptivity**. The osteoarthritic joint is a hostile biochemical environment: acidic, oxidative, protease‐rich, and cytokine‐laden (Yao et al. 2023; Tóth et al. 2021). This milieu can compromise vesicles, alter their surface properties (including protein‐corona effects), and render target cells refractory to pro‐repair cues (Tóth et al. 2021; Hu et al. 2021). Inflammatory mediators such as interleukin‐1β and tumor necrosis factor‐α suppress chondrogenesis, induce apoptosis, and tilt synovial macrophages toward catabolic phenotypes; oxidative stress can compromise nucleic acid cargo; proteases can cleave membrane proteins important for uptake (Yao et al. 2023; Tóth et al. 2021; Hu et al. 2021). Therapeutic messages are not delivered into silence; they are delivered into a noisy conversation dominated by disease signals. Short, mechanism‐guided “priming” pharmacologic anti‐inflammatory regimens, orthobiologic adjuncts, or mechanical unloading may be required to make the joint receptive (Yao et al. 2023).

Viewed through these axes, the preclinical‐clinical discrepancy is not a paradox but an expected outcome when potency, precision, and persistence are not jointly optimized. These four axes cargo, surface, kinetics, and context define what a human study must measure; the next question is whether our trial design and endpoints read the right signals.

To translate these principles into a clinically interpretable framework, we summarize how specific OA endotypes map onto EV design strategies, delivery approaches, and measurable outcomes (**Table** **2**).

**Table 2:** Endotype‐guided EV therapy and expected outcomes in osteoarthritis.

OA Endotype	Dominant pathology	Recommended EV design	Delivery approach	Biomarkers to monitor	Expected measurable outcome
Inflammatory OA	Synovitis, macrophage activation	Anti‐inflammatory MSC‐EVs (miR‐146a‐like cargo)	Intra‐articular ± repeat dosing	Synovial cytokines, macrophage markers	Pain improvement, ↓ synovitis MRI
Cartilage‐dominant OA	Matrix degradation	Chondroprotective EVs (miR‐140‐5p enriched)	Cartilage‐targeted EVs or depot	Cartilage turnover markers (COMP, CTX‐II), T2 mapping	Slower cartilage loss
Bone‐remodelling OA	Subchondral bone lesions	Bone‐targeting EVs	Repeat dosing / retention systems	Bone marrow lesion imaging	Structural stabilization
Metabolic OA	Oxidative stress, lipotoxicity	Mitochondrial‐protective EVs	Systemic or IA repeat dosing	Oxidative stress markers	Functional improvement

*Note*: Representative experimental and translational studies supporting these associations are discussed in the relevant sections above.

### 3.3 Trial Design and Endpoint Mismatch

Trial design in OA remains a critical determinant of whether the therapeutic signal of EVs can be detected or obscured. Current studies are fragmented by unstratified enrolment that mixes inflammatory, cartilage‐dominant, bone‐remodelling, and metabolic phenotypes, each with distinct pathogenic drivers and distinct probabilities of responding to vesicle therapy. Without enrichment, true responders are diluted within heterogeneous cohorts, leading to inconclusive outcomes (Hunter and Bierma‐Zeinstra 2019). Because OA biology is temporally dynamic, stratification should be considered longitudinal rather than a single baseline assignment (Hunter and Bierma‐Zeinstra 2019; Mobasheri et al. 2017; Kraus et al. 2017). A patient classified within an inflammatory‐dominant state early in disease may later transition toward cartilage‐structural or subchondral bone‐remodelling processes, altering both therapeutic responsiveness and measurable outcomes (Hunter and Bierma‐Zeinstra 2019; Mobasheri et al. 2017). Accordingly, biomarker and imaging readouts should be incorporated as repeated measures to monitor target engagement and mechanism‐specific effects (Kraus et al. 2017). Endpoint interpretation should therefore emphasize biological response and trajectory modification rather than relying solely on fixed clinical scores at a single timepoint. Standardization of patient selection and stratification based on reproducible biomarkers such as synovial‐fluid cytokine panels, compositional MRI of cartilage, or EV‐cargo transcriptomic signatures would provide objective anchors for trial design (Witwer et al. 2019; Kraus et al. 2011). Similarly, endpoints must extend beyond subjective pain indices (e.g., Western Ontario and McMaster Universities Osteoarthritis Index [WOMAC]) to incorporate quantitative imaging (delayed gadolinium‐enhanced magnetic resonance imaging of cartilage [Dgemric], T2 mapping), dynamic subchondral perfusion metrics, and biochemical readouts of matrix turnover. Embedding such objective measures early in trial design will be essential to generate reproducible, comparable data across studies and to establish potency–response relationships that can guide regulatory approval. These challenges are compounded by the choice of endpoints, many of which have historically lagged behind mechanism. Importantly, the proposed precision framework should not be interpreted as requiring simultaneous implementation of all technologies in every trial. Rather, it represents a hierarchical translational strategy. Early mechanistic studies in specialized centres may incorporate multi‐omic profiling, advanced imaging, and intensive pharmacodynamic monitoring to establish biological proof‐of‐mechanism. Once target engagement and responsive patient groups are identified, later‐phase trials and real‐world studies can employ simplified biomarker panels, routine imaging, and standardized clinical outcomes. In this way, complex tools function as developmental instruments to de‐risk therapy rather than permanent requirements for clinical deployment, allowing EV therapeutics to remain compatible with diverse healthcare infrastructures.

**Endpoints have likewise lagged behind mechanism**. Radiographic joint space width is relatively insensitive on the time horizons and effect sizes plausible for first‐in‐class vesicles (Hunter and Bierma‐Zeinstra 2019; Tang et al. 2025; Roemer et al. 2020). Pain‐dominant outcomes such as WOMAC are clinically necessary but can be vulnerable to placebo unless paired with objective biology (Hunter and Bierma‐Zeinstra 2019). Quantitative MRI that registers compositional change in cartilage and bone marrow lesions, validated measures of synovitis (contrast‐enhanced MRI; positron emission tomography [PET] where appropriate), and prespecified pharmacodynamic panels in synovial fluid and serum can confirm that the intended pathway was modulated in the same participants who report symptomatic change (Roemer et al. 2020; Guermazi et al. 2011; Roemer et al. 2011). Embedding serial sampling of synovial fluid EVs and their cargo can offer a direct window into whether exogenous therapeutic messages have shifted the endogenous EV network (Welsh et al. 2024). Accordingly, WOMAC/KOOS (Knee Injury and Osteoarthritis Outcome Score) should be paired with objective imaging endpoints that read on‐target biology within trial timeframes.

### 3.4 Box | Outcomes beyond WOMAC: Imaging Indicators to Pair With EV Mechanisms

**Cartilage composition (qMRI)**. Use T2/T1ρ mapping and/or dGEMRIC to quantify matrix water and proteoglycan loss‐compositional MRI is sensitive to early cartilage change and recommended in modern reviews of OA imaging (Eckstein et al. 2021; Link et al. 2023). For dGEMRIC specifically, feasibility/responsiveness in (pre)radiographic knee OA has been shown, including pilot randomized controlled trial (RCT) data (Mcalindon et al. 2011; Tiderius et al. 2003).

**Synovitis burden (contrast‐enhanced MRI)**. CE‐MRI segmentation of synovial tissue volume (STV) provides a quantitative, treatment‐responsive target that relates to symptoms; STV has been proposed and validated as a treatment target in symptomatic knee OA (O'Neill et al. 2016; O'Neill et al. 2016). A recent radiology review summarizes practical CE‐MRI options when steroids/anti‐inflammatories are under study (Thoenen et al. 2022).

**Bone‐marrow lesions (BMLs)**. MRI‐detected BML size/volume/severity are associated with pain and structural progression and can change on month‐scale horizons appropriate as mechanistic co‐primary/secondary for subchondral‐dominant endotypes (Sofat and Howe 2024; Walsh et al. 2023).

**Subchondral bone turnover (PET/MRI)**. ^18F NaF PET/MRI quantifies subchondral bone perfusion/mineralization; it has been used in OA knees (including dynamic and exercise‐response protocols) and correlates with clinical measures in recent studies use as a PD marker in bone‐remodelling endotypes (Ziegeler et al. 2025; Watkins et al. 2022).

**Design note**. Choose imaging endpoints to match EV mechanism (e.g., cartilage‐anabolic EVs → T2/T1ρ/dGEMRIC; anti‐synovitis EVs → CE‐MRI STV; bone‐targeted EVs → BMLs and ^18FNaF PET). Contemporary OA imaging state‐of‐the‐art reviews provide planning ranges and protocol considerations.

**Trial design details matter**. Injection vehicle and needle gauge can influence intra‐articular distribution; rescue‐medication policies and physical‐therapy co‐interventions introduce variance; stratification by alignment and meniscal status reduces biomechanical confounding. Adaptive designs can accelerate learning about dose and schedule while preserving blinding and control; umbrella (master‐protocol) frameworks can test multiple EV constructs across endotypes in parallel. The common thread is mechanistic read‐through: a trial should be built to tell us not only whether pain changed, but whether the biology we targeted actually moved.

### 3.5 Manufacturing Heterogeneity and the Missing Potency Compass

Manufacturing variability is the quiet driver of clinical inconsistency. EV preparations differ by source tissue and donor, parent‐cell passage, culture media and supplements, oxygen tension, harvest timing, and isolation method (ultracentrifugation, SEC, tangential‐flow filtration, precipitation, affinity capture). Each parameter reshapes both cargo and surface properties (Welsh et al. 2024; van Niel et al. 2018; Bertolino et al. 2023). Analytical characterization that stops at particle counts, size distributions, and a handful of tetraspanins is often orthogonal to therapeutic effect and invites batch‐to‐batch drift (Welsh et al. 2024; Bertolino et al. 2023).

A credible chemistry‐manufacturing‐controls (CMC) framework for EV therapeutics in OA should therefore move beyond enumeration toward mechanism‐anchored critical quality attributes. Multi‐omic cargo fingerprints (microRNA, protein, and lipid composition) need to be aligned to validated human cell–based potency assays: synoviocyte NF‐κB reporter suppression, chondrocyte anabolism and autophagy rescue under inflammatory stress, osteoclast resorption‐pit assays and osteoblast mineralization readouts to capture subchondral remodelling (Welsh et al. 2024; Bertolino et al. 2023). These assays should be performed under standardized conditions and linked statistically to in vivo activity so that release specifications reflect function, not just identity. From a regulatory perspective, this shift from identity and enumeration to mechanism‐anchored critical quality attributes (CQAs) is not cosmetic; it is prerequisite for credible dose justification, lot comparability, and multi‐site trials (Welsh et al. 2024; Bertolino et al. 2023). Potency as a release criterion, stability that is potency‐indicating (not merely size‐indicating), and predefined comparability protocols for process changes are the elements that convert a promising vesicle preparation into a product that can advance through clinical phases. Stability studies must quantify potency retention across storage and thaw cycles, given that freeze‐thaw can induce vesicle aggregation or cargo degradation that is invisible to particle counters but can be devastating to efficacy (Bertolino et al. 2023). Process analytical technologies in‐process monitoring of conductivity, protein carryover, and vesicle integrity during tangential‐flow filtration or chromatography should be integrated to reduce lot‐to‐lot variability. Finally, reference standards and cross‐site proficiency testing are essential to make results portable between centres and trials.

Beyond potency and comparability, an equally critical yet underappreciated dimension of EV manufacturing is long‐term stability and the feasibility of off‐the‐shelf formulations. Cryopreservation preserves vesicle integrity but can alter cargo release kinetics and bioactivity, whereas lyophilization combined with cryoprotectants such as trehalose or sucrose offers long‐term stability and facilitates global distribution (Witwer et al. 2019; Lener et al. 2015). Comparative studies indicate that optimized freeze‐drying can maintain EV morphology, RNA cargo, and immunomodulatory potency, enabling room‐temperature transport and stockpiling. These advances make it increasingly realistic to envision EVs as stable, distributable biologics, moving the field closer to true off‐the‐shelf therapies. Embedding stability testing and comparability protocols into early‐phase trials will be essential to align regulatory expectations and guarantee reproducible potency across manufacturing sites. To operationalize these points, the following checklist summarizes manufacturing, stability, and formulation choices for an off‐the‐shelf EV product.

### 3.6 Box | off‐the‐Shelf EVs: Manufacturing, Stability, and Formulation

**Upstream/Downstream (GMP‐amenable)**. Use closed culture (e.g., 3D/hollow‐fibre or stirred bioreactor) and a tangential‐flow filtration (TFF) → SEC purification train to reduce protein aggregates and scale reproducibly; report process variables per MISEV2023 (pre‐analytics, separation, characterization, and potency) and include a mechanism‐anchored potency assay at release (Welsh et al. 2024; Monguió‐Tortajada et al. 2019; Bae et al. 2024).

### 3.7 Stability Reagents & Storage Conditions (Liquid)

- **Buffers**: isotonic PBS/HEPES with disaccharides (e.g., trehalose 25–100 mM) to limit aggregation/ice injury; avoid repeated freeze–thaw cycles.
- **Conditions**: −80°C gives best retention of size/marker integrity; −20°C and 4°C degrade faster; short‐term 4°C (≤1–2 weeks) only if a cryo‐protectant is present and potency is re‐checked (Ahmadian et al. 2024; Lyu et al. 2022).
- **‘Liquid EV’ versus ‘Powder EV’ (lyophilized)**. Lyophilization with trehalose (and, where appropriate, mild surfactant) preserves colloidal stability and bioactivity after drying and enables refrigerator shipping; on reconstitution, verify particle count/size and potency. −80°C frozen liquid remains the gold standard for long‐term banking; lyophilized is attractive for distribution and clinic‐side storage when validated by stability‐indicating assays (Lyu et al. 2022; Golan and Stice 2024; Susa et al. 2023).

### 3.8 Formulate “Pure EVs” or Combine with Materials?

For intra‐articular (IA) use, co‐formulation in hyaluronic‐acid (HA) or other hydrogels extends residence time, protects vesicles, and can improve functional readouts versus free EVs. Practical options include HA‐based or thermoresponsive depots that shear‐thin through a needle and re‐gel in situ. Report polymer MW, %w/v, and rheology (Diaz‐Rodriguez et al. 2021; Wang et al. 2022; Zhao et al. 2022).

### 3.9 Evidence That Evs & Ha Can Work Together

Preclinical IA studies show EVs with HA (or sequential EV & HA dosing) increase joint retention and sustain cartilage repair signals over weeks compared with free EV boluses. Use this to justify an induction & maintenance schedule in trials (Wong et al. 2020).

### 3.10 Shelf‐Life Expectations (to State in CMC)

Bank frozen master lots at −80°C with stability testing (size/markers and potency at 0, 3, 6, 12 months). For lyophilized presentations, justify a 4–12‐week refrigerator claim (longer if you generate real‐time/accelerated data). Always pair physical QC (quality control) with biological potency (e.g., NF‐κB reporter suppression, chondrocyte anabolism rescue) (Ahmadian et al. 2024; Lyu et al. 2022).

### 3.11 Can We Use Peptides to Enhance EV Secretion?

Yes, but treat as process development, not default GMP. Up‐regulating nSMase2 activity increases small‐EV release (ceramide pathway) and manipulating syntenin–ALIX/Rab axes can also boost secretion; however, these can alter cargo and require comparability data. Prefer bioreactor/process optimization first; explore secretagogue strategies only with strict characterization (Teng and Fussenegger 2021; Choezom and Gross 2022; Debbi et al. 2022).

A related blind spot is immunogenicity and off‐target biodistribution. Although EVs are generally well tolerated, repeated dosing over months in large joints warrants systematic assessment of anti‐vesicle antibodies, complement activation, and uptake by non‐target tissues. These are not reasons to slow development; they are reasons to bring analytical rigor commensurate with a therapeutic class that sits between biologics and nanoparticles (van Niel et al. 2018; Bertolino et al. 2023; Hoshino et al. 2015).

### 3.12 Synthesis: Principles That Fall Out

When the preclinical record is parsed and the clinical experience is placed on the mechanistic axes of cargo, surface, kinetics, and context, the field's problem becomes operational rather than conceptual. Therapeutic EVs falter in humans not because the idea is naïve, but because we routinely underdeliver signal strength, signal specificity, and signal persistence into joints whose disease networks are resistant to change. The remedy is consequently specific and testable (Tang et al. 2025; Bertolino et al. 2023; Roemer et al. 2020; Kraus et al. 2024).

**First, treat EVs as programmable biologics**. Specify cargo compositions that neutralize dominant pathways in defined endotypes anti‐inflammatory miRNA sets and IL‐1 pathway antagonism for synovitis‐heavy disease; cargo that restrains osteoclastogenesis and stabilizes osteoblast function for bone‐remodelling endotypes; miRNAs that support SRY‐box transcription factor 9 (SOX9) signalling, autophagy, and survival for cartilage‐dominant disease; stress‐response and lipid‐handling programs for metabolic OA. Engineer surfaces with cartilage‐ or bone‐homing ligands to increase uptake by deep‐zone chondrocytes and subchondral units, rather than assuming diffusion will suffice at human scale (Tang et al. 2025; Bertolino et al. 2023; Geiger et al. 2018; Bajpayee and Grodzinsky 2017).

**Second, solve pharmacokinetics**. Human knees require residence‐time technologies, for example, cartilage‐penetrating carriers and mechanically resilient intra‐articular depots (e.g., shear‐thinning or thermoresponsive hydrogels) that prolong exposure; where depots are impractical, plan repeat dosing a priori and justify schedule using early PD markers rather than arbitrary intervals. Exposure must be designed, not hoped for (Geiger et al. 2018; Bajpayee and Grodzinsky 2017; Joshi et al. 2025).

**Third, make the joint receptive**. Short‐course anti‐inflammatory priming, mechanical unloading and alignment strategies, and metabolic optimization can reduce the biochemical headwind into which therapeutic messages are delivered. Sequencing matters: calm the joint first; then deliver the regenerative signal (Hunter and Bierma‐Zeinstra 2019; Tang et al. 2025).

**Fourth, read the right signal**. Pair clinical outcomes with mechanistic endpoints capable of moving on trial timescales: quantitative MRI for cartilage composition (T2, T1ρ, dGEMRIC) and bone marrow lesions; validated measures of synovitis (contrast‐enhanced MRI; PET where appropriate); and prespecified PD panels in synovial‐fluid and serum to show that the intended pathway was modulated in the same participants who report symptomatic change. Embed serial profiling of endogenous EVs and their cargo to test the central hypothesis that exogenous therapeutic vesicles can shift the resident EV network (Welsh et al. 2024; Roemer et al. 2020; Kraus et al. 2024).

**Fifth, manufacture to function**. Build CMC systems around mechanism‐anchored potency assays and multi‐omic fingerprints; institute stability and comparability protocols that track function across lots and over time; and adopt shared reference materials so data across centres are commensurable. Regulators do not require perfection; they require clarity about what matters and evidence that it is controlled (Welsh et al. 2024; Bertolino et al. 2023).

**These principles yield testable predictions**. In an inflammatory‐dominant endotype, an EV construct whose cargo, surface, and exposure are tuned should demonstrate early suppression of synovitis on imaging and cytokines in synovial fluid, followed by improvements in pain and function; failure to see the biological changes should trigger prompt adaptation of dose, schedule, or delivery rather than prolonged pursuit of symptomatic endpoints alone. Conversely, in bone‐remodelling endotypes, the earliest success signal should appear in bone marrow lesion size and intensity with secondary improvements in cartilage composition; absence of these shifts suggests that the surface logic or cargo selection requires revision. Across endotypes, translational success should be defined by convergence: imaging, biomarkers, and symptoms moving in the directions predicted by mechanism (Tang et al. 2025; Roemer et al. 2020; Kraus et al. 2024).

In short, the EV paradox is less a barrier than a design brief. The preclinical–clinical discordance will narrow as products move from “vesicles made in a particular way” to vesicles that accomplish a particular biological task in a particular endotype, delivered in a manner that secures persistence in human joints. When potency, precision, and persistence are engineered together and verified by on‐target biology the experimental signal should reappear in the clinic, not as isolated anecdotes but as reproducible, disease‐modifying effects.

## 4 Part III. A Precision Medicine Roadmap for EV Therapeutics in OA

### 4.1 Endotyping the Patient to Match the Therapy

OA comprises overlapping mechanistic endotypes prominently inflammatory, bone‐remodelling, cartilage‐dominant, and metabolic rather than a single uniform disease (Tang et al. 2025; Angelini et al. 2022). A precision strategy should therefore define who is most likely to benefit from a given EV construct by pairing clinical phenotype with mechanistic biomarkers. For inflammatory‐dominant OA, use synovial‐fluid cytokine panels (e.g., IL‐1β, TNF‐α) and emerging exo‐/transcriptomic signatures that report immune pathway activity; layer imaging readouts such as quantitative MRI of synovitis and contrast‐enhanced synovial tissue volume, with PET where available, to map active inflammation (O'Neill et al. 2016; Thoenen et al. 2022; Moulin et al. 2025). For bone‐remodelling endotypes, combine MRI BML mapping with subchondral perfusion/turnover metrics For example, dynamic contrast‐enhanced MRI parameters and ^18FNaF PET indices of bone metabolism (Jena et al. 2022; Watkins et al. 2021). For cartilage‐dominant disease, apply compositional cartilage MRI (T2, T1ρ, dGEMRIC) as pharmacodynamic markers, alongside serum/urine cartilage‐turnover biomarkers such as COMP and CTX‐II (used with FNIH‐established reference intervals) (Mosher 2025; Li et al. 2024; Kraus et al. 2017). For metabolic endotypes, incorporate adipokine panels and lipidomic signatures reflecting metabolic‐inflammation coupling (Wei et al. 2023; Berenbaum et al. 2017). Single‐cell atlases of OA joint tissues and multi‐omic risk models can further refine stratification by identifying pathway activation states (e.g., NF‐κB, Wnt, TGF‐β/BMP) in specific compartments (synovium, fat pad, cartilage, subchondral bone) (Liu et al. 2025; Nielsen et al. 2024). In practice, an endotyping algorithm should be prospectively embedded in trials to enrich for patients whose dominant drivers are biologically countered by an EV's designed mechanism, and to anchor mechanism‐linked pharmacodynamic endpoints.

**Figure 4:**
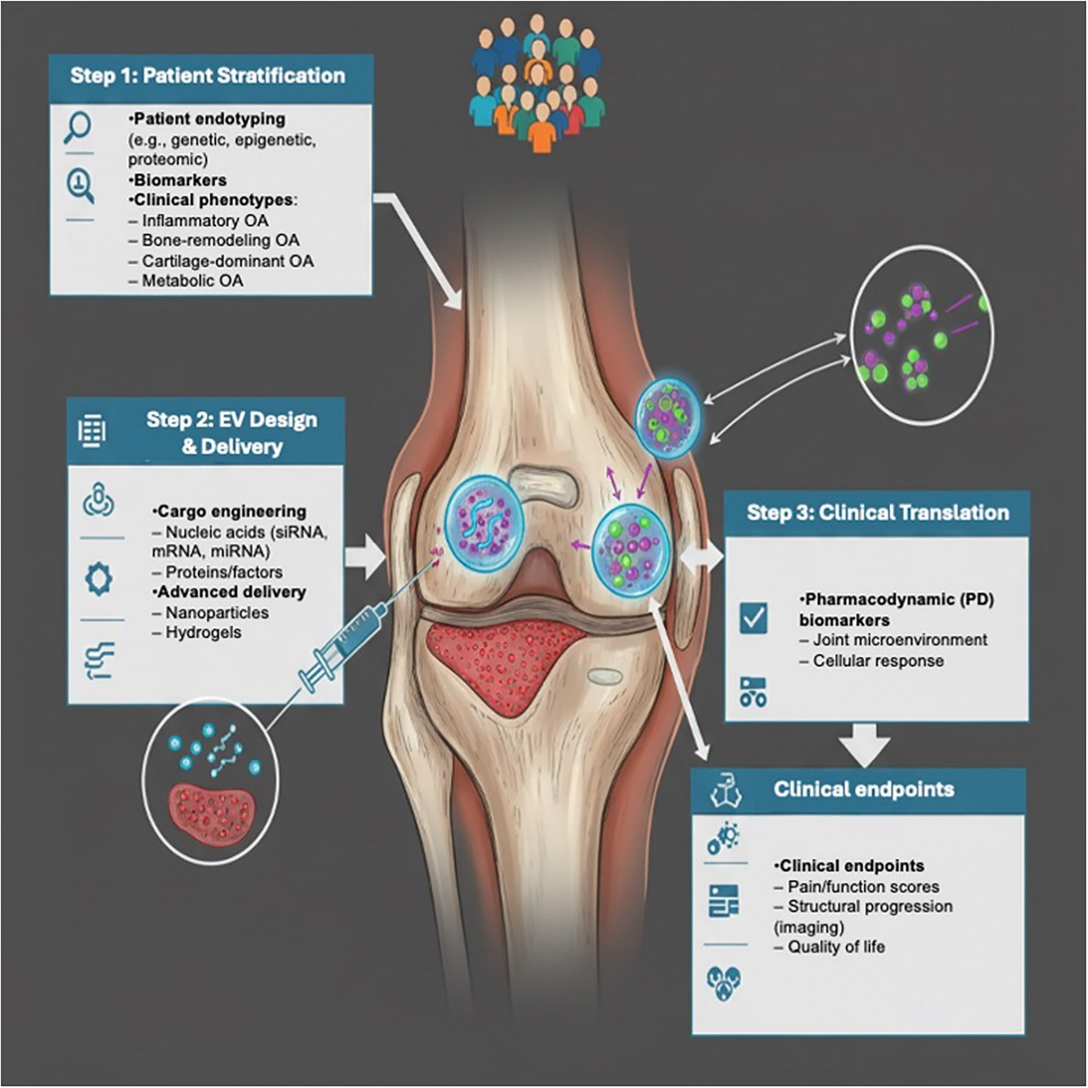
Precision medicine roadmap: from endotype to outcome. A comprehensive framework for extracellular vesicle (EV) therapeutics in osteoarthritis, beginning with patient endotyping and biomarker‐driven stratification, followed by rational EV design and advanced delivery systems, and culminating in clinical translation with pharmacodynamic biomarkers and validated endpoints.

This stepwise strategy from patient endotyping to tailored EV design, advanced delivery, and mechanistically anchored endpoints can be conceptualized as a precision medicine roadmap (**Figure** **4**).

### 4.2 Engineering EVs for Potency and Specificity

Therapeutic EVs must deliver instructions strong enough and precise enough to counter the resident pathogenic network. Potency is primarily a function of cargo logic; targeting is governed by surface biology.

The choice of parent cell source is a primary determinant of EV cargo composition, particularly miRNA profiles, and thereby dictates therapeutic potency. For example, synovial MSC‐derived EVs are enriched in miR‐140‐5p, a cartilage‐protective miRNA that directly supports matrix regeneration in knee OA (Tao et al. 2017). Infrapatellar fat pad MSC‐EVs contain high levels of miR‐100‐5p and show cartilage‐protective activity in vivo (Wu et al. 2019). Beyond tissue‐resident MSC and chondrocyte sources, pluripotent cell‐derived EVs offer a broader immunomodulatory profile with emerging evidence in OA models. By contrast, iPSC‐derived EVs appear to carry a broader repertoire of regenerative and anti‐inflammatory miRNAs, although detailed characterization of their cargo in OA models remains limited. Current studies demonstrate that iPSC‐EVs suppress pro‐inflammatory cytokine release, protect chondrocytes, and attenuate cartilage degradation, suggesting that their therapeutic effects are mediated more by global immunomodulatory activity than by enrichment of a single miRNA species (Hsueh et al. 2023; Zhu et al. 2017). Chondrocyte‐derived EVs carry lineage‐specific cargo such as miR‐95‐5p, which enhances cartilage matrix synthesis (Mao et al. 2018). Collectively, these examples underscore that selecting the optimal parental cell type is not merely a manufacturing decision but a biological lever to enrich for specific reparative miRNAs tailored to osteoarthritic pathology.

**Inflammatory‐dominant endotypes**. EVs enriched for microRNAs that down‐tune Toll‐like receptor (TLR)–NF‐κB signalling and reprogram macrophages toward reparative states are rational. For example, synovial fibroblast–derived EVs overexpressing miR‐146a suppress TRAF6/TLR4–NF‐κB signalling, reduce cartilage damage, and shift macrophages from M1 to M2 in OA models (Wang et al. 2024). For chondro‐protection and matrix anabolism, miR‐140‐5p loading is supported by robust preclinical data showing miR‐140‐EVs preserve cartilage and prevent OA progression (Tao et al. 2017). When protein antagonists are desired (e.g., IL‐1 pathway blockade), contemporary designer exosome platforms enable stable protein/nucleic‐acid loading and surface retargeting (Kojima et al. 2018; Alvarez‐Erviti et al. 2011).

**Bone‐remodelling endotypes**. Crosstalk between subchondral bone and cartilage is EV‐mediated: osteocyte EV cargo (e.g., miR‐23b‐3p) can drive chondrocyte catabolism and OA progression in vivo, establishing a tractable target axis (Liu et al. 2025). Accordingly, EV cargo sets that restrain osteoclastogenesis and normalize osteoblast activity are appropriate, paired with bone‐targeting ligands to increase delivery to subchondral units (Wang et al. 2023; Xu et al. 2023; Sun et al. 2016).

**Cartilage‐dominant disease**. Chondro‐anabolic programs remain central. EVs enriched with miR‐140‐5p (cartilage homeostasis) demonstrate cartilage protection and OA prevention in rats (Tao et al. 2017). Parent‐cell preconditioning can further sharpen cargo: TGFβ3‐primed MSC‐EVs (rich in miR‐455) promote chondro‐protection and regeneration, offering a practical route to reproducible, mechanism‐anchored EV composition (Sun et al. 2022).

**Metabolic endotype (actionable design)**. Obesity‐associated OA features low‐grade metaflammation, lipotoxic stress, and mitochondrial dysfunction. A rational EV construct is to reverse this triad by: (i) anti‐miR‐34a to derepress the sirtuin 1 (SIRT1)/PGC‐1α axis (miR‐34a directly suppresses SIRT1 in OA chondrocytes and promotes apoptosis/senescence (Yan et al. 2016); PGC‐1α supports chondrocyte mitochondrial resilience (Wang et al. 2023); (ii) KEAP1 siRNA / Nrf2‐stabilizing cargo to enhance antioxidant programs and blunt ROS‐driven cartilage injury (Nrf2 activation reduces cartilage breakdown and OA features in vivo (Davidson et al. 2013); Nrf2 declines with age in joint tissues (Taylor et al. 2023); and (iii) a minimal set of metaflammation‐modulating miRNAs (e.g., miR‐146a at resolution‐favouring doses) to down‐tune TLR–NF‐κB signalling in synovium and infrapatellar fat pad (see above). Early pharmacodynamic anchors can include leptin/adiponectin ratios, CRP, targeted lipidomics (ceramides), and synovial‐fluid cytokines, paired with MRI of IFP inflammation and cartilage composition (Kloppenburg et al. 2025).

**Surface logic to put dose where biology lives**. Decorating vesicles with cartilage‐homing motifs increases deep‐zone chondrocyte uptake; the collagen II‐binding peptide WYRGRL boosts intra‐cartilage retention and penetration in vivo (shown across OA nanocarriers (Xue et al. 2021): comprehensive EV example with dual‐engineered/cartilage‐targeted EVs: open‐access study detailing WYRGRL utility for EVs (Liu et al. 2023)). Mechanistically analogous exosome targeting via peptide‐Lamp2b fusions is well‐validated (Alvarez‐Erviti et al. 2011), and recent applications fuse WYRGRL–Lamp2b for cartilage targeting in vivo (Yuan et al. 2024). For synovium/IFP bias, CD44‐interacting HA coatings can preferentially engage CD44‐high joint stromal and synovial cells; in OA joints, HA‐nanoparticles demonstrate CD44‐dependent retention and disease‐relevant activity (Kang et al. 2021).

**Manufacturing levers**. Parent‐cell preconditioning (e.g., TGFβ3) and genetic programming (e.g., EXOtic devices) reproducibly tune EV cargo and yield (Kojima et al. 2018; Sun et al. 2022).

### 4.3 Overcoming Pharmacokinetics With Residence‐Time Technologies

Human joints are large, mobile compartments with brisk lymphatic and vascular clearance; single boluses of unmodified intra‐articular (IA) therapies EVs included rarely sustain target engagement for long enough to change disease biology. Extending joint residence time and tuning release kinetics are therefore central to EV drug design in OA. Authoritative pharmacokinetic reviews and translational studies converge on this point: IA agents are rapidly eliminated from synovial fluid, distribution into cartilage is limited, and formulations that slow clearance (microspheres, hydrogels, nanoparticles) improve local exposure while reducing systemic spillover (Siefen et al. 2022; Kraus et al. 2018; Bruno et al. 2022; Gao et al. 2022). Hydrogel depots (shear‐thinning or thermoresponsive) are a practical first line to prolong EV exposure. Hyaluronan‐based systems add biocompatibility and lubrication, and have repeatedly shown that entrapped EVs/sEVs can be released in a controlled fashion intra‐articularly with superior chondroprotection versus free EVs. Examples include Diels–Alder‐crosslinked HA/PEG hydrogels delivering MSC small EVs, and HA/Pluronic thermogels that keep chondrocyte‐derived EVs in the joint and release them for ∼2 weeks while improving macrophage polarization and cartilage scores in OA models (Yang et al. 2021; Kalairaj et al. 2024; Sang et al. 2022). Localization to failing cartilage can be engineered with collagen‐binding motifs so the depot “sticks” where it matters. Collagen‐hybridizing peptides (CHPs) that anneal to denatured collagen markedly extend intra‐articular retention in vivo after IA injection, and peptides that bind type II collagen (e.g., WYRGRL) have been used to bias uptake to cartilage and enhance efficacy of OA nanotherapies design rules that can be ported to EV surfaces or EV‐bearing scaffolds (Xue et al. 2021; Luke et al. 2023). Chitosan‐based nanoparticles and composites are biodegradable, cationic carriers that can further slow clearance and aid cartilage interaction. In OA models, chitosan platforms have improved joint retention and chondroprotection; a notable high‐impact example conjugated chitosan to the chondroanabolic small molecule kartogenin and achieved durable IA efficacy (Kang et al. 2014; Kou et al. 2019). Thermoresponsive in‐office depots (e.g., PLGA–PEG–PLGA or micelle‐to‐gel systems) are injected as liquids and form gels in situ, enabling sustained release over weeks in large joints. This class is well‐documented across cartilage‐disease delivery reviews and preclinical IA studies and is compatible with EV loading or co‐loading strategies (Bruno et al. 2022; Yi et al. 2023; Rouco et al. 2025).

Where depot formation is impractical (certain trial designs or joint anatomies), repeat‐dose regimens should be specified a priori and guided by joint volume and expected clearance kinetics, with pharmacodynamic anchors (e.g., synovial cytokines, MRI readouts) confirming sustained target engagement rather than peak exposure. This dosing logic follows directly from joint PK principles and clinical translation experience (Siefen et al. 2022; Gao et al. 2022).

Terminology for delivery materials is standardized in Box 1.

### 4.4 Box 1 | Delivery Materials: Definitions Used in This Review

- **Hydrogel**: A water‐swollen, cross‐linked polymer network (continuous phase) that retains large amounts of water. Examples include natural‐polymer hydrogels such as hyaluronan (HA), collagen, chitosan, and synthetic hydrogels such as poly(vinyl alcohol) (PVA), poly(acrylic acid) (PAA), and poly(*N*‐isopropylacrylamide) (PNIPAM) (a thermoresponsive system that flows on injection and gels in situ near body temperature) (Li and Mooney 2016; Jenkins and Little 2019).
- **Scaffold**: A three‐dimensional (often porous/fibrous) solid support intended to integrate with tissue and provide mechanical context/anchorage (distinct from a continuous gel network). Example: a collagen‐mimetic scaffold positioned at a cartilage defect (Jenkins and Little 2019).
- **Nanoparticle (NP)**: A discrete colloidal carrier (typically tens to a few hundred nanometres) used for loading and controlled release (e.g.,, chitosan nanoparticles). NPs are not hydrogels unless cross‐linked into a continuous gel matrix (Blanco et al. 2015).

**Usage in this Review**. We say, “HA hydrogel” (gel network), “collagen‐mimetic scaffold” (solid support), and “chitosan nanoparticles” (discrete colloids). When combined, we write “collagen‐mimetic scaffold with an HA‐hydrogel overlay”.

**Figure 5:**
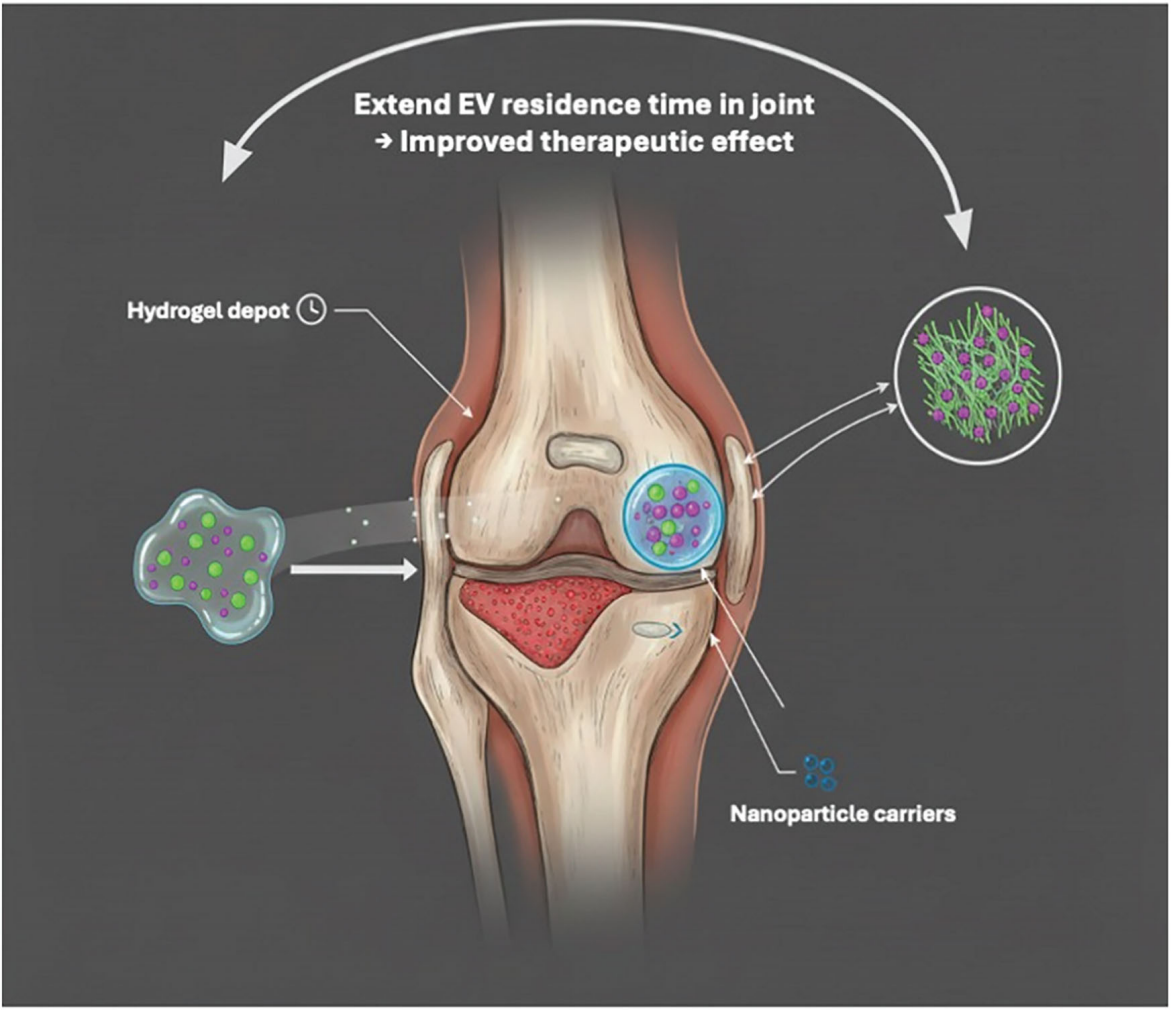
Delivery systems overview. Cartoon illustrating hydrogel depots and nanoparticle carriers as advanced strategies to extend the residence time of extracellular vesicles (EVs) within the joint microenvironment, thereby improving therapeutic efficacy.

Residence‐time technologies such as hydrogels and nanoparticle carriers provide rational solutions to prolong intra‐articular exposure and enhance EV efficacy (**Figure** **5**).

However, these delivery platforms are not without potential limitations. Biomaterial depots may provoke local inflammatory or foreign‐body responses depending on polymer composition, crosslinking chemistry, and degradation products. Encapsulation can also alter vesicle release kinetics or membrane interactions, and excessive retention or adsorption to carrier materials may reduce effective EV bioavailability and biological activity. From a translational perspective, hydrogel viscosity, injectability through clinical needle gauges, and batch‐to‐batch material reproducibility represent practical constraints, particularly for intra‐articular administration in large human joints. In addition, scalable manufacturing of combination products (EVs plus biomaterial carriers) introduces regulatory complexity, as both the vesicle product and the delivery material must meet quality and safety specifications. Accordingly, delivery systems should be interpreted not as universally beneficial enhancements, but as context‐dependent pharmacokinetic tools whose advantages must be balanced against manufacturability and clinical practicality.

### 4.5 Conditioning the Joint to Receive a Regenerative Signal

Even optimally engineered EVs struggle in an unreceptive milieu. Short, mechanism‐guided conditioning can improve receptivity: brief anti‐inflammatory priming to reduce synovitis, mechanical unloading/alignment to decrease focal stress, and metabolic optimization when systemic drivers dominate. Evidence‐based OA guidelines support weight loss/exercise and short‐term intra‐articular glucocorticoids for flare‐level synovitis (Bannuru et al. 2019; Kolasinski et al. 2020). MRI data show synovial tissue volume falls after IA steroids, aligning with a “calm first, then regenerate” sequence (O'Neill et al. 2016; Thoenen et al. 2022). Unloader bracing can reduce pain and improve function in medial compartment disease (Thoumie et al. 2018). Platelet‐derived products remain mixed; if used as a biologic primer, composition should be defined and complementary to the EV cargo, and expectations should reflect RCT heterogeneity (negative high‐quality RCTs alongside positives) (Belk et al. 2021; Bennell et al. 2021).

### 4.6 Manufacturing Discipline and Potency Analytics

Clinical credibility hinges on consistency and functional potency, not particle counts alone. Align release with MISEV2023 best‐practice characterization (pre‐analytics, separation, storage, functional readouts) and modern GMP/CMC thinking for EV therapeutics (Welsh et al. 2024). Define CQAs mapped to mechanism (e.g., NF‐κB suppression in human synoviocytes; chondro‐anabolic rescue under IL‐1β) and qualify a reference standard for cross‐lot normalization. For scalable, closed‐system processing, TFF supports GMP‐compatible concentration/purification at volume (Busatto et al. 2018). Stability programs should specify storage and thaw conditions and justify excipients; storage parameters and freeze–thaw cycles materially affect EV integrity and function (Welsh et al. 2024; Gelibter et al. 2022).

### 4.7 Trial Architecture That Reads the Right Signal

When residence‐time technologies are integral (e.g., EV & hydrogel depot), evaluate the combination as the clinical product from phase I onward. Enrich by endotype (see earlier section) and choose primary endpoints aligned to mechanism and timescale: quantitative MRI for cartilage composition and bone‐marrow lesions, validated measures of synovitis, and prespecified PD biomarkers in synovial fluid/serum (including EV‐cargo PD) to confirm target engagement (Kraus et al. 2017; Mcalindon et al. 2015). Pain/function (e.g., WOMAC) remain necessary but should be paired with biological readouts to deconvolute placebo effects. Adaptive/umbrella designs within OARSI trial‐design principles can accelerate learning across endotypes and dosing schedules, with early futility boundaries to avoid under‐informative studies.

Translating these design principles into a practical framework requires linking patient endotypes with tailored EV constructs, delivery platforms, and readouts an approach operationalized in Box 2.

### 4.8 Box 2 | Operationalizing Precision: From Patient Endotype to EV Construct and Endpoints

**Inflammatory‐dominant**

*Biomarkers*: Synovial‐fluid IL‐1β/TNF‐α↑; qMRI/PET synovitis volume↑; EV‐cargo inflammatory signature (NF‐κB).

*EV design*: Anti‐inflammatory cargo: miR set that restrains NF‐κB (e.g., miR‐146a); protein/siRNA payloads that antagonize IL‐1 pathways (e.g., IL‐1Ra). Surface: synovium‐tropic motifs (e.g., CD44‐interacting hyaluronan fragments) to bias uptake by synoviocytes/macrophages.

*Delivery*: HA hydrogel depot for intra‐articular retention; PNIPAM thermoresponsive hydrogel for in‐situ gelation and sustained release; repeat‐dose schedule pre‐specified.

*PD readouts*: SF cytokine drop (IL‐1β, TNF‐α); shift in synovial EV‐cargo from pro‐ to anti‐inflammatory; macrophage polarization markers.

*Clinical endpoints*: WOMAC/KOOS (pain & function) paired with qMRI synovitis change as a mechanistic co‐primary.

**Bone‐remodelling**

*Biomarkers*: qMRI BML↑ (size/intensity); subchondral perfusion abnormalities; bone turnover markers.

*EV design*: Anti‐osteoclastogenic cargo: miR/protein set that reduces RANKL–NFATc1 signalling; osteoblast‐stabilizing factors. Surface: bone‐targeting ligands (e.g., hydroxyapatite‐affine moieties/bisphosphonate‐conjugated peptides).

*Delivery*: Thermoresponsive PNIPAM hydrogel or collagen‐mimetic scaffold seated at the osteochondral unit; optional chitosan nanoparticles for depot‐style release.

*PD readouts*: BML metrics (size, ΔT2*), bone turnover panels; subchondral perfusion normalization; SF markers of bone–cartilage crosstalk.

*Clinical endpoints*: Pain/function plus BML response on qMRI (co‐primary); KOOS‐ADL as supportive.

**Cartilage‐dominant**

*Biomarkers*: Compositional cartilage MRI T2/T1ρ/dGEMRIC abnormal; serum/urine COMP, CTX‐II ↑.

*EV design*: Chondro‐anabolic cargo: miR‐140‐5p enrichment; factors supporting SOX9, autophagy and survival; optional TIMP‐like anti‐catabolic payloads. Surface: collagen‐binding motif to enhance deep‐zone chondrocyte uptake.

*Delivery*: Collagen‐mimetic scaffold at defect for localization, optionally overlaid with HA hydrogel; schedule to maintain exposure over remodelling cycles.

*PD readouts*: MRI cartilage composition shift (ΔT2/ΔT1ρ/ΔdGEMRIC); SF matrix turnover markers; chondrocyte stress‐response panels.

*Clinical endpoints*: KOOS subscales (Pain/Symptoms/Function) with cartilage composition change as mechanistic co‐primary; structural responder index as secondary.

**Metabolic**

*Biomarkers*: BMI↑, leptin/adiponectin↑, CRP↑; lipidomics (ceramides↑); MRI IFP inflammation.

*EV design*: Metabolic‐reprogramming cargo: anti–miR‐34a to de‐repress SIRT1/PGC‐1α; KEAP1 siRNA to stabilize Nrf2 antioxidant axis (↑SOD2/catalase); small pro‐resolution miRNA panel (e.g., miR‐146a). Surface: CD44‐tropic motif to bias delivery to synovium/IFP stromal cells.

*Delivery*: PNIPAM thermoresponsive hydrogel for sustained residence; or HA hydrogel plus chitosan nanoparticles as a composite depot; dosing aligned to systemic drivers.

*PD readouts*: Serum CRP, leptin/adiponectin ratio; targeted lipidomics (ceramides); SF cytokines; MRI IFP inflammation and cartilage composition.

*Clinical endpoints*: WOMAC/KOOS with metabolic biomarker panel and IFP‐imaging change as mechanistic co‐primary; compositional MRI as key secondary.

### 4.9 Combination Logic Without Therapeutic Sprawl

OA is multifactorial, but combination therapy should stay principled. Rational pairs include: short anti‐inflammatory or unloading priming followed by endotype‐matched EVs; EVs plus a biomaterial depot to extend intra‐articular exposure; and EVs combined with mechanical interventions (e.g., gait retraining or valgus‐unloader bracing) that reduce local pathogenic load at the delivery site. Combinations that redundantly hit the same pathway or that add complexity without measurable mechanistic gain should be avoided; each component should raise the probability of crossing one or more limiting thresholds in humans (potency, precision, or persistence) (Van De Looij et al. 2023; Uhlrich et al. 2025; Gueugnon et al. 2021; Pritchard et al. 2013).

### 4.10 Regulatory and Ethical Considerations

As EV therapeutics mature, clarity on product definition matters: vesicles alone versus vesicles integrated with a depot (e.g., hydrogel) can change regulatory classification and quality expectations. When a depot is integral to performance, early scientific advice and treating the combination as the clinical product from phase I onward can prevent reclassification surprises. For nanoscale biologics like EVs, biodistribution, immunogenicity, and off‐target uptake require systematic evaluation using assays suitable for EVs (e.g., quantitative PET/SPECT radiolabels; validated fluorescence/bioluminescence methods with awareness of dye pitfalls). Release specifications should be potency‐linked and anchored to the intended mechanism (e.g., suppression of synovial NF‐κB programs, chondro‐anabolic rescue), not merely particle counts (Welsh et al. 2024; Van De Looij et al. 2023; Rai et al. 2024; Lázaro‐Ibáñez et al. 2021; Khan et al. 2022).

### 4.11 Trial Architecture and Equity

Enrich early trials by endotype (to read on‐target biology), but pair this with explicit equity safeguards so access isn't narrowed to advantaged subgroups. Adaptive and master‐protocol designs (umbrella/platform) can accelerate learning across endotypes and dosing schedules while enabling early transition of non‐responders to alternatives; pairing clinical endpoints with mechanistic biomarker readouts helps deconvolute placebo effects (Precision Medicine Needs an Equity Agenda 2021; Pallmann et al. 2018; Woodcock and LaVange 2017). Although some elements of this framework, such as adaptive trial structures, advanced imaging biomarkers, and pharmacodynamic monitoring, may appear resource‐intensive, their implementation need not be uniform across healthcare settings. A tiered translational approach is more realistic: early mechanistic trials in specialized centres can employ comprehensive imaging (e.g., quantitative MRI or PET) and biomarker panels to establish biological proof‐of‐mechanism, whereas later‐phase or real‐world studies may rely on simplified surrogate measures such as compositional MRI, validated clinical scores, and limited laboratory biomarkers (Kloppenburg et al. 2025). Similarly, short priming regimens and repeat‐dose schedules can be incorporated into standard orthopaedic or rehabilitation workflows without requiring highly specialized infrastructure. From a regulatory and trial‐design perspective, such stepwise adoption is consistent with master‐protocol and adaptive design principles used to improve efficiency and interpretability across heterogeneous patient subgroups (Woodcock and LaVange 2017), while acknowledging that advanced therapies also face non‐uniform regulatory and access pathways across regions, which can shape feasibility and implementation (Izeta and Cuende 2023). Thus, the proposed strategies should be viewed as scalable translational options rather than mandatory components of every clinical implementation environment.

## 5 Conclusions and Outlook

OA remains stubbornly without a disease‐modifying therapy not because the field lacks plausible biology, but because our interventions have not yet been designed to contend with the joint's entrenched, system‐level pathology. EVs crystallize this challenge. In OA, vesicular communication is not an epiphenomenon; it encodes the disease itself. Endogenous EVs propagate inflammatory and catabolic programmes, while therapeutic or engineered EVs can, in principle, deliver the inverse signal. The EV paradox simultaneously pathogenic and therapeutic offers a unifying lens through which the disconnect between striking preclinical results and modest clinical outcomes becomes legible.

Viewed through that lens, clinical shortfalls are not mysterious. Human joints are large, chronically inflamed, and efficiently drain intra‐articular agents; single bolus injections of unmodified vesicles rarely provide sufficient potency, precision or persistence to outcompete the background flux of pathogenic EVs. Heterogeneous endotypes dominant synovitis, bone‐remodelling, cartilage attrition, or metabolic drivers further ensure that a generic product aimed at a generic population will deliver diluted signals. At the same time, manufacturing variability and the absence of mechanism‐anchored potency assays mean that two “EV doses” can differ by orders of magnitude in functional strength, undermining dose justification and reproducibility. Importantly, current human studies demonstrate safety and feasibility but have not yet shown consistent structural improvement or durable modification of OA progression, underscoring the need for cautious interpretation of preclinical efficacy.

A credible way forward is now visible. First, diagnose the joint by endotype rather than by radiograph alone, and enrol accordingly. Secondly, treat EVs as programmable biologics: specify cargo that neutralizes the dominant pathway in each endotype, and engineer surfaces that home to the right micro‐anatomical niche deep‐zone cartilage, synovium or subchondral bone. Thirdly, solve pharmacokinetics with residence‐time technologies and rational repeat‐dosing, so that therapeutic messages are present long enough to reset cellular states. Fourthly, pair clinical outcomes with mechanistic readouts compositional MRI, synovial and serum pharmacodynamic biomarkers, EV‐cargo signatures to confirm target engagement and to separate biology from placebo. Finally, institutionalize manufacturing discipline: adopt multi‐omics fingerprints and human cell‐based potency assays as release criteria, and build closed, scalable processes that make the same product every time.

If executed together, these elements convert the EV paradox from a roadblock into a design constraint. Meaningful evaluation should extend beyond pain outcomes and include biological indicators such as synovitis, bone marrow lesion dynamics, and cartilage matrix turnover, which would provide evidence supporting disease modification if observed. Equally important is clarity about failure modes we can now avoid single‐dose, non‐targeted injections into unstratified cohorts; reliance on particle counts and tetraspanins as surrogates for potency; and trials powered on insensitive structural endpoints without mechanistic confirmation.

There are broader implications for the field. Establishing shared reference standards for EV analytics, common potency assays tied to OA mechanisms, and open data resources linking EV composition to pharmacodynamics would accelerate learning and comparability across centres. Platform or umbrella trials that randomize within endotypes could shorten the path from signal detection to dose optimization, while preserving ethical equipoise by transitioning non‐responders early. Attention to access and scalability from donor sourcing to cost‐aware bioprocessing will be essential to ensure that “precision” does not become a synonym for “scarcity”.

EV therapeutics are not a panacea; OA will continue to demand integrated care that addresses biomechanics, comorbidity and behaviour alongside molecular repair. But the conceptual shift advocated here treating OA as an EV‐mediated network disorder and treating EVs as programmable counter‐signals provides a disciplined framework within which durable disease modification is plausible. With endotype‐guided trial design, controlled manufacturing, and mechanism‐anchored endpoints, EVs may be rigorously tested as a potential disease‐modifying strategy for OA.

Field‐level call to action. We propose a precompetitive, global collaboration to establish (i) mechanism‐anchored potency assays using human synoviocytes, chondrocytes, and osteoclast/osteoblast co‐cultures; (ii) shared reference EV lots with multi‐omic fingerprints; and (iii) a minimal reporting standard that couples analytics to prespecified pharmacodynamic readouts in early trials. Harmonized methods and open resources would accelerate learning across centres, improve regulatory confidence, and shorten the path from signal detection to dose optimization. The EV paradox then becomes not a barrier but the design brief for a new therapeutic class in OA (Hunter and Bierma‐Zeinstra 2019; Tang et al. 2025; Bertolino et al. 2023; Geiger et al. 2018).

## Author Contributions

**Duc‐Hiep Bach**: conceptualization, supervision, writing – original draft, writing – review and editing. **Thanh Liem Nguyen**: conceptualization, writing – original draft, writing – review and editing, supervision. **Van Giang Bui**: methodology, validation.

## Disclosure

D.‐H.B. used ChatGPT (OpenAI) and Gemini (Google) as language‐support tools during manuscript preparation to assist with organization and clarity. These tools were also used to generate preliminary conceptual figure drafts based on the authors. scientific descriptions. They were not used as sources of scientific authority. All hypotheses, interpretations, references, and conclusions were independently developed and verified by the authors, who take full responsibility for the content of the manuscript.

## Ethics Statement

Not applicable. This review synthesizes previously published studies assnd did not involve new studies with human participants or animals.

## Consent

The authors have nothing to report

## Conflicts of Interest

The authors declare no conflicts of interest.

## Data Availability

No new datasets were generated or analysed for this manuscript. All data discussed are from previously published sources cited in the article.
